# Plasma exosome miRNA-26b-3p derived from idiopathic short stature impairs longitudinal bone growth via the *AKAP2/ERK1/2* axis

**DOI:** 10.1186/s12951-023-01849-8

**Published:** 2023-03-16

**Authors:** Xijuan Liu, Jinghong Yuan, Zhiwen Wu, Junqiu Zhang, Yunfeng Shen, Jingyu Jia

**Affiliations:** 1 Department of Pediatrics, The Second Affiliated Hospital of Nanchang University, Nanchang City, Jiangxi Province China; 2 Department of Orthopedics, The Second Affiliated Hospital of Nanchang University, Nanchang City, 330006 Jiangxi Province China; 3 Endocrine Department, The Second Affiliated Hospital of Nanchang University, Nanchang City, Jiangxi Province China

**Keywords:** Idiopathic short stature, Endochondral ossification, Exosomes, microRNA, miR-26b-3p, Bone growth

## Abstract

**Background:**

Currently, the etiology of idiopathic short stature (ISS) is still unclear. The poor understanding of the molecular mechanisms of ISS has largely restricted this strategy towards safe and effective clinical therapies.

**Methods:**

The plasma exosomes of ISS children were co-cultured with normal human chondrocytes. The differential expression of exosome miRNA between ISS and normal children was identified via high-throughput microRNA sequencing and bioinformatics analysis. Immunohistochemistry, In situ hybridization, RT-qPCR, western blotting, luciferase expression, and gene overexpression and knockdown were performed to reveal the key signaling pathways that exosome miRNA of aberrant expression in ISS children impairs longitudinal bone growth.

**Results:**

Chondrocytes proliferation and endochondral ossification were suppressed after coculture of ISS plasma exosomes with human normal chondrocytes. High-throughput microRNA sequencing and RT-qPCR confirmed that plasma exosome miR-26b-3p was upregulated in ISS children. Meanwhile, exosome miRNA-26b-3p showed a high specificity and sensitivity in discriminating ISS from normal children. The rescue experiment showed that downregulation of miR-26b-3p obviously improved the repression of chondrocyte proliferation and endochondral ossification caused by ISS exosomes. Subsequently, miR-26b-3p overexpression inhibited chondrocyte proliferation and endochondral ossification once again. In situ hybridization confirmed the colocalization of miR-26b-3p with AKAP2 in chondrocytes. In vitro and in vivo assay revealed exosome miRNA-26b-3p impairs longitudinal bone growth via the AKAP2 /ERK1/2 axis.

**Conclusions:**

This study is the first to confirm that miR-26b-3p overexpression in ISS plasma exosomes leads to disorders in proliferation and endochondral ossification of growth plate cartilage via inhibition of AKAP2/ERK1/2 axis, thereby inducing ISS. This study provides a new research direction for the etiology and pathology of ISS and a new idea for the biological treatment of ISS.

**Graphical Abstract:**

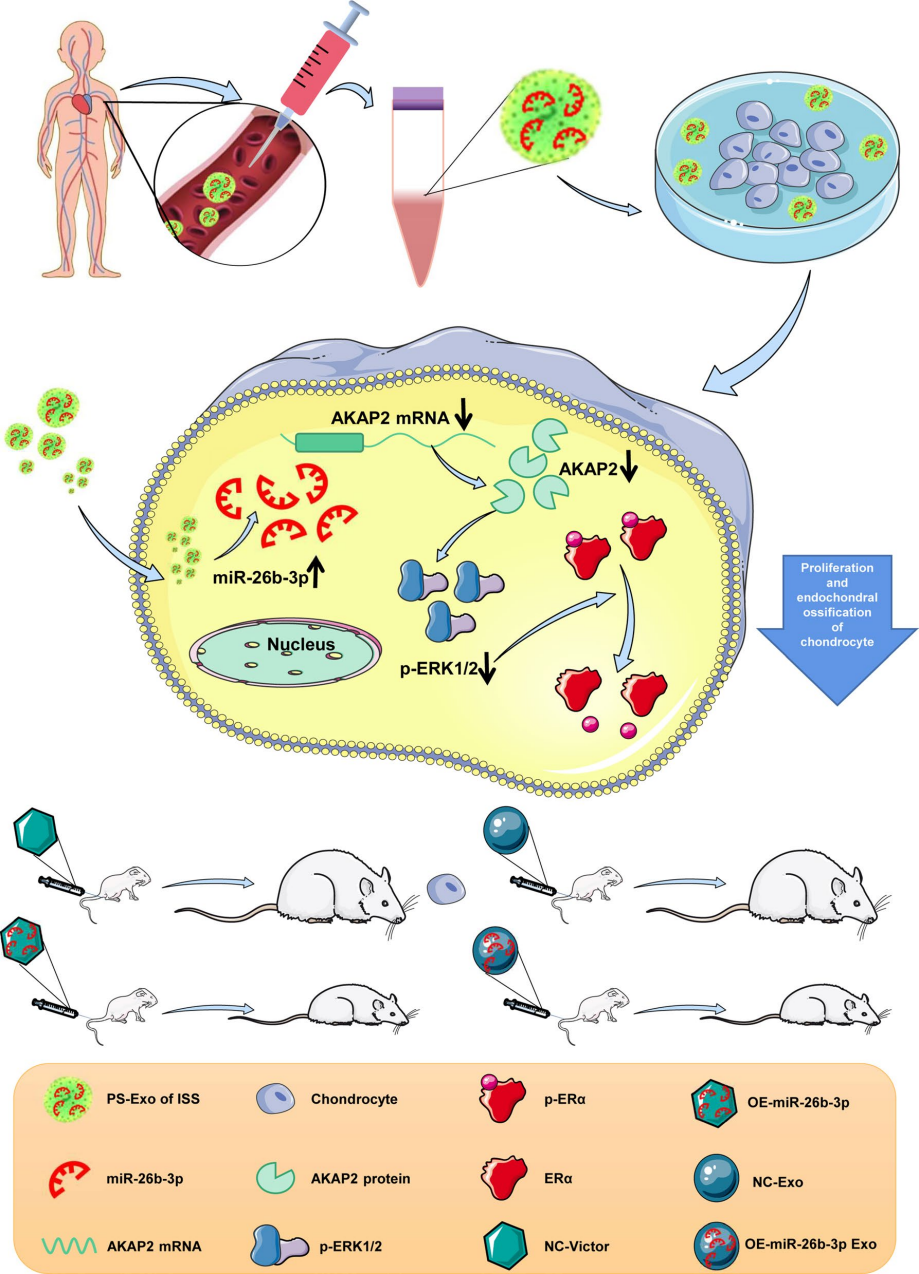

**Supplementary Information:**

The online version contains supplementary material available at 10.1186/s12951-023-01849-8.

## Introduction

Short stature refers to individuals whose height is below the mean height by more than 2 standard deviations or below the 3rd percentile of the normal population in the same race, sex, age, and similar living circumstances [1]. Short stature has adverse effects on the individual’s future learning, work, life, and psychology. Both anxiety and depression scores in short stature groups were reported to be higher than those in the control group [2]. The use of recombinant growth hormone opens up excellent therapeutic prospects for individuals who are short-statured. However, less than 1% of cases of idiopathic short stature (ISS) in children result from growth hormone deficiency. The remaining children were diagnosed with ISS after excluding chronic systemic diseases, skeletal or endocrine abnormalities, nutritional deficiencies, and chromosomal abnormalities. These children had a peripheral blood growth hormone provocation peak > 10 μg/L. The prevalence of short stature in adolescents ranges from 0.64%–3.77%, in which ISS accounts for 30–80% [3]. Currently, the etiology of ISS is unclear, and effective diagnostic and therapeutic measures are lacking.

Human height is regulated by an interactive interplay of genetic makeup and external environmental factors. Genetic factors play an important role in influencing the height of an individual. Recently, whole-genome scan technology has been used to screen for mutations and to perform association analyses of ISS patient genomes and revealed that GHR, SHOX, FGFR3, ACAN, and NPR2 are related to the incidence of ISS [4–9]. Although these studies have made important contributions to elucidate the etiology of ISS, the mutation rate of the above genes is approximately 5% in individuals with ISS. For instance, the mutation rate is approximately 1.4% for ACAN [7], 2–4% for SHOX [5], approximately 5% for GHR [4], and approximately 6% for NPR2 [8]. Therefore, there is no effective marker suitable for the early diagnosis and screening of ISS. As opposed to growth hormone-deficient short stature, individuals with ISS do not lack growth hormone. To increase the final height of pediatric patients with ISS, clinicians mainly use recombinant human growth hormone. It has been demonstrated that the long-term use of growth hormone to treat ISS can improve the final height. However, the efficacy and safety of growth hormone therapy is still controversial. Previous studies have reported that recombinant human growth hormone can effectively increase the adult height in individuals with ISS in a dose-dependent manner; i.e., a high dose is better than a low dose [9–11]. Nevertheless, Van et al*.* [12] found no significant differences in the final height between individuals treated with the high-dose recombinant human growth hormone versus the controls. Additionally, considering the role of the growth hormone-insulin-like growth factor (IGF) endocrine axis in regulating cell growth and proliferation and its role in tumor pathogenesis, the clinical safety of recombinant human growth hormone in treating ISS has also attracted extensive attention. Carel et al*.* [13] followed up 6500 children with ISS, growth hormone deficiency, or those diagnosed small for gestational age, who were treated with recombinant human growth hormone. The results demonstrated that the incidence of bone or cartilage tumors and cerebrovascular diseases in these children were higher than that in the normal population. In another study, 6700 French patients diagnosed with ISS and treated with recombinant human growth hormone exhibited a significantly higher incidence of intracerebral hemorrhage compared with the control [14]. Stochholm et al*.* [15] reported that treatment using growth hormone did not increase the incidence of stroke and tumor but increased the incidence of type 2 diabetes in comparison with the control. Currently, therapy using recombinant human growth hormone shows significant heterogeneity in the treatment of ISS. Moreover, the safety of the long-term use of recombinant human growth hormone requires further evaluation. According to the 2016 guidelines of the American Pediatric Endocrinology Association, not all individuals diagnosed with ISS are suitable candidates for treatment with recombinant human growth hormone [16]. Silvers et al*.* [17] pointed out that there were potentially more than 500,000 individuals with ISS in the United States and that the annual cost of recombinant human growth hormone treatment was approximately US$10 billion. In China, the average treatment cost of ISS is also expected to be 100,000 RMB; however, the therapeutic benefit and outcome is uncertain. Therefore, clarification of the pathogenesis of ISS based on molecular biology is urgently needed to diagnose ISS, identify its specific markers, and achieve effective treatment outcomes.

Growth plate chondrocytes are cartilage tissue with growth potential that are sandwiched between epiphysis and metaphysis. They constitute the main differentiation area of longitudinal bone growth, which is known as the “engine” of childrens’ growth and development and determine the longitudinal growth rate of bone and the final height of the individual. Several hormones and growth factors secreted by tissues and organs are delivered to growth plate chondrocytes by blood, which acts as a carrier. These hormones and factors interact with growth plate chondrocytes and regulate growth and development through cell communication. Studies have shown that there was no abnormal secretion of growth hormone, glucocorticoid, thyroid hormones, and insulin growth factor in the circulating blood of individuals with ISS. In addition to hormones and growth factors, the circulating blood is enriched with exosomes. Exosomes are small vesicles actively secreted by cells. They have a lipid bilayer membrane and are 30–100 nm in diameter. Exosomes express markers such as CD63, CD9, CD81, and MHC.1 on their surface and contain proteins and nucleic acids [18–20]. As a key tool of intercell communication, exosomes play a role in intercellular material transport and information transmission. Several types of cells can secrete exosomes to deliver proteins, mRNA, miRNA, and other substances to recipient cells. Moreover, exosomes regulate cell behavior and play an important role in many physiological and pathological processes such as tumor metastasis, immune recognition, and inflammatory responses [21–26]. Additionally, exosomes can also be used as natural nanoparticles for drug delivery. Exosomes can not only be used as an early diagnostic marker for several diseases but also as a carrier of drugs that are targeted to specific locations for disease treatment. Many studies [27–29] have shown that plasma exosome miRNA can be used as an early diagnostic marker and a targeted treatment tool for diseases, thereby highlighting its wide application prospects. However, the role of exosomes in the pathogenesis of ISS has not been reported. In this study, we identified the differential expression of plasma exosome miRNA in ISS and reveal its role in the pathogenesis of ISS.

## Materials and methods

### Patient information

In this study, we enrolled 70 patients with ISS treated in our hospital from October 2016 to March 2020 and 70 healthy children examined in our hospital during the same period. The average age of patients with ISS was 8.92 ± 0.34 years, with a range of 4–12 years. Their average height was 120.2 ± 1.72 cm, with a range of 88.71–142.2 cm. There were 31 males and 39 females among the healthy children with an average age of 8.39 ± 0.28 years and a range of 4–12 years. Their average height was 132.1 ± 1.57 cm, with a range of 103.9–150.9 cm. All individuals were diagnosed with ISS according to the definition of ISS [16, 30, 31], height below the mean height of more than 2 standard deviations or below the 3rd percentile of the normal population in the same race, sex, age, and similar living circumstances. All participants have normal growth hormone levels and normal birth weight, without short stature caused by endocrine, nutritional, or systemic disorders, and without skeletal dysplasia, chromosome abnormalities, or psychological and emotional disorders.

The blood samples of subjects from the ISS and control groups used for the study were discarded blood samples from laboratory examination. Samples were obtained and immediately frozen with liquid nitrogen and stored at –80℃ until further use. Five pairs of blood samples were used for miRNA chip analysis. All blood samples were used for RT-qPCR to detect miRNA expression. Informed consent from the family members was obtained. The ethics committee of our hospital approved the study protocols.

### Differential expression analysis of exosome miRNA

The total exosome RNA of all samples was extracted and purified using the exoRNeasy serum/plasma MIDI Kit (QIAGEN, GmbH, Germany) according to the manufacturer’s protocol. Ten samples were used to analyze miRNA differential expression in exosome between ISS children and normal children. RNA quality was assessed based on RNA integrity, which was detected using an Agilent Bioanalyzer 2100. Total RNA was amplified using rolling circle replication. According to the instructions provided, cRNA was labeled with biotin and purified using a Low Input Quick Amp Labeling Kit (Agilent Technologies, Santa Clara, CA, US) and RNeasy small kit (QIAGEN, GmBH, Germany). cRNA labeled with cy3 was connected to the microarray using a Gene Expression Hybridization Kit (Agilent Technologies, USA) at 65 °C and 41 h in the hybridization box and washed after hybridization. Scanning of microarrays and acquisition and data analysis were performed using Agilent’s Micro whole column scanner (dye channel, green; scan resolution, 3 m; PMT, 100%; bit depth, 20 bits) and Feature Extraction Software 12.0. The score bit method of the limma package in R language was used to normalize chip data and screen and compare the differentially expressed miRNAs from the ISS and control groups. Fold change ≥ 2.0 and P < 0.05 (Student’s *t*-test) were set as the threshold for screening.

### Bioinformatics analysis and prediction of target genes

TargetScan (http://www.targetscan.org/) was used to predict the downstream target genes of miR-26b-3p. Cytoscape (http://www.cytoscape.org/) was used to visualize the miRNA-mRNA network and describe the results. Gene ontology (GO; http://www.geneongoloty.org/) and DAVID 6.8 (https://david.ncifcrf.gov/) were used for GO and Kyoto Encyclopedia of Genes and Genomes (KEGG) analysis. P < 0.05 was set as the threshold.

### Culture of chondrocytes

Chondrocytes were provided by Procell Life Science Technologies. Dulbecco’s Modified Eagle Medium (DMEM; Gibco, Thermo Fisher Scientific, Inc.) with 10% fetal bovine serum (Gibco, Australia) and 1% penicillin/streptomycin was used as the culture medium. The incubation conditions were 37 °C in an atmosphere of 5% CO_2_.

### Analysis of cell proliferation

The effects of miR-26b-3p on chondrocyte proliferation were investigated using cell counting kit-8 (CCK-8). For the CCK-8 assay, cultured chondrocytes were seeded into 96-well plates (2000 cells/well). CCK-8 (10 μL) was added to each well after incubation for 24 h, 48 h, 72 h, or 96 h at 37 °C in an atmosphere of 5% CO_2_. Absorbance was detected at 450 nm using a microplate reader (VARIOSKAN FIASH, Agilent, USA) after incubation for 2 h.

### RT-qPCR and western blotting

Total RNA was extracted from chondrocytes using Trizol reagent (Invitrogen, USA) according to the manufacturer’s instructions. RNA was reverse-transcribed to cDNA using a PrimeScriptTM RT Reagent kit with gDNA Eraser and PCR (ABI Q6). Primers of miR-26b-3p, AKAP2, collagen type X, osteocalcin (OCN), Osteopontin (OPN), RUNX2, ERKI/2, and ER-α are listed in the Additional file 1: Table S1. The reaction conditions were as follows: 95 °C for 10 min for pre-denaturation, 95 °C for 10 s, 60 °C for 34 s, and 40 cycles for extension. The relative RNA expression was obtained using the 2^−ΔΔCt^ method, which reflected the relative expression levels of target genes in the ISS group. GAPDH was used as a control. Three replicates were performed for each experiment.

Cultured chondrocytes were lysed with RIPA lysis buffer (Applygen Technologies Inc.) to isolate total protein, and the concentration was determined using the bicinchoninic acid method. Proteins were separated using polyacrylamide gel electrophoresis (Beijing biosynthesis Biotechnology) and transferred to polyvinylidene fluoride membranes. The membranes were blocked using nonfat milk (Solarbio Inc.) for 2 h at room temperature and incubated overnight at 4 °C with the primary antibody. Next, they were washed three times with 1 × TBST and incubated with rabbit-anti-mouse secondary antibody (Abcam, USA) for 1 h. ImageJ was used to analyze the gray values of protein bands after development. The following antibodies were used for immunoblotting: anti-AKAP2 (1:1000, CST, USA), anti-RUNX2 (1:1000; Abcam, USA), anti-COL10A (collagen type X, 1:1000; Abcam, USA), anti-OPN (1:1000; Abcam, USA), anti-OCN (1:3000; Abcam, USA), anti-ERK1/2 (1:3000; Abcam, USA), anti-p-ERK1/2 (1:3000; Abcam, USA), ER-α (1:3000; Abcam, USA), and p-ER-α (1:3000; Abcam, USA). GAPDH (1:1000, Abcam, USA) and β-actin (1:1000, Abcam, USA) were used as an endogenous control. We used the WB Stripping buffer (NO:21059; Thermo Fisher SCIENTIFIC, USA) to re-probe the membrane with a different primary antibody if more than one protein was detected. A gray value is then calculated by Image Lab (v 5.2.1).

### Luciferase expression

Plasmids containing miR-26b-3p wild-type 3′ untranslated region or miR-26b-3p mutant 3′ untranslated region and AKAP2 mimics were cotransfected into HEK 293 T using LipoFiter TM 3.0 transfection reagent (Hanbio Biotechnology, China). Double-luciferase activity was measured using the double-luciferase reporter assay system (Promega Corporation) after 48 h of transfection.

### Alkaline phosphatase (ALP) activity

ALP assay was performed following the manufacturer’s instructions in the kit (Solarbio Inc.). BCIP/NBT chromogenic reagent was added after washing the fixed chondrocytes and incubated for 20 min in the dark. Then, nuclear fast red staining solution was added and allowed to react for a further 5 min for counterstaining. When the color development was as expected, distilled water was used to terminate the reaction. Images were acquired using microscopy.

### Overexpression vector of miRNA and overexpression of siRNA and AKAP2

MiR-26b-3p plasmids were constructed from the Shanghai Genechem Co.,Ltd. Cell transfection was performed using Lipofectamine 3000 (Invitrogen, Carlsbad, USA) following the instructions. Before 24 h of transfection, 2 × 10^5^ cells/well were seeded in six-well plates and cultured in complete medium without penicillin/streptomycin. Lipofectamine 3000 (50 μL of opti-MEM + 1 μL Lipo3000) and plasmids (50 μL opti-MEM + 1 μg DNA + 2 μL P3000) were diluted with opti-MEM. The cell suspensions were mixed and placed for 20 min at room temperature. Next, 100 μL of the mixture and 400 μL opti-MEM were added to each well and incubated at 37 °C in an atmosphere of 5% CO_2_ for 6 h. The infection efficiency was determined using microscopy. Lastly, the medium was changed to DMEM containing serum and incubated for 72 h. AKAP2 was overexpressed using recombinant AKAP2 protein (Novus, Bio-Techne, USA). siRNAs (miR-26b-3p siRNA, NC-miRNA siRNA, AKAP2 siRNA, and NC-mRNA siRNA) were transfected using Lipofectamine 2000 (Invitrogen, Carlsbad, USA). Lipofectamine 2000 (50 μL opti-MEM + 1 μL Lipo2000) and siRNA (50 μL opti-MEM + 5 μL siRNA) were diluted using opti-MEM. The cells were incubated for 72 h for the next experiment.

### Preparation of miRNA loaded exosomes

MiRNA loaded exosomes were constructed using ExoFectin^®^ sRNA-into-Exosome Kit (P401). The miRNA-26 sequence is 5′CCGGGACCCAGUUCAAGUAAUUCAGGAUAGGUUGUGUGCUG.

UCCAGCCUGUUCUCCAUUACUUGGCUCGGGGACCGG synthesized by Guangzhou RiboBio Co., Ltd. (China). NC mimics (sense UUUGUACUACACAAAAGUACUG and antisense CAGUACUUUUGUGUAGUACAAA) were obtained from Guangzhou RiboBio Co., Ltd. (China). According to the manufacturer’s instructions, ExoFectin suspension (Supplemental buffer 25 µl and ExoFectin Reagent 5 µl) and miRNA suspension (Supplemental buffer 25 µl and miRNA (10 µM) ~ 1 µl) were prepared. ExoFectin suspension (30 µl) and miRNA suspension (~ 26 µl) were mixed. Incubate at room temperature for 5 min. 50 µg exosomes were added into miRNA-ExoFectin complex, and were mixed well by gentaly pipet up and down, and were incubated at 37 °C for overnight. Harvest the transfected exosomes using PureExo^®^ Kit (Cat.#: P101). The exosome is transfected (loaded with siRNA) and ready for the downstream experiment.

### Internalization of exosomes by human chondrocytes

To confirm whether the chondrocytes could take up exosomes derived from chondrocytes, exosomes and miRNA were labelled using green fluorescent lipophilic dye (Vybrant DiO) and yellow represents exosomes and miR-26-3p transferred cells respectively. Human chondrocytes were then incubated for 6 h with exosomes derived from labelled cells. After washing with PBS, the DiO-labelled exosomes were observed in the perinuclear region of the chondrocytes (Fig. 6A), confirming the internalization by chondrocytes.

### High-throughput mRNA sequencing

mRNA sequencing was performed on an Illumina HiSeq X Ten sequencer. A TruSeq Stranded mRNA LT Specimen Prep Kit (Illumina, San Diego, CA, USA) was used to construct the mRNA library. Data were optimized and dimeric, nonsense, and low complexity sequences were removed using Trimmomatic. Sequence alignment for RNA-seq was performed using Hisat2. For alignment results, the specific fragments per kilobase of transcript per million mapped reads values were exported using Cufflink. Transcriptome reads were counted using htseq-count. Differential expression analysis was performed using DESeq in the R program. Lastly, GO and KEGG analyses were performed at a threshold of P < 0.05 and fold change > 2.

### microRNA high-throughput sequencing

Total RNA extraction of ISS plasma was conducted using xoRNeasy Plasma Midi Kit (Cat #0.77044, Qiagen). Then the RNA was reverse-transcribed according to the manufacturer’s protocol and high-throughput sequencing was performed using a NextSeq 500 sequencer (Illumina, San Diego, CA, USA). The raw sequences were preprocessed by the FASTXToolkit (fastx_toolkit-0.0.13.21) to flter out the low-quality reads, as well as sequences with 3′ primers, with 5′ primer, with polyA tails, or with insert tags. To obtain clean reads, contaminant sequences were removed from raw reads prior to length distribution analysis. Unique reads (15–40 nt) were extracted from clean reads by discarding repeated sequences and following BLAST alignment against the Rfam database version 11.0 (Rfam, Cambridge, UK). Known rRNA, tRNA, snRNA, and snoRNA sequences were not considered as unique reads. The remaining unique read sequences were further subjected to BLAST in order to screen for conservative miRNAs using the miRBase database version 21.0 (miRBase, Manchester, UK). Differential expression of conservative miRNAs was considered only when the fold change was ≥ 2 and p value < 0.05 using the DESeq R package version 1.18.0 (Bioconductor, Seattle, WA, USA). This work was performed by Shanghai Biotechnology Corporation (Shanghai, China).

### Bioinformatics

The miRNA candidate targets were predicted using miRanda microRNA Target Detection Software (http://www.microRNA.org.) and TargetScan (http://www.targetscan.org/vert_71/). Meanwhile, analyses were conducted using the Gene Ontology project (GO; July 01, 2019 release; Open Biomedical Ontologies, USA) and Kyoto encyclopedia of genes and genomes (KEGG; Kyoto University, Kyoto, Japan). GO analysis comprised a series of bio logical process (BP), cellular component (CC), and molecular function (MF) assessments. The identified miRNA targets were subjected to GO enrichment analysis according to their corresponding mRNAs. The significance levels of GO terms among differentially expressed genes were evaluated by the enrichment scores derived from -log10 (p-value). To assess the role of target genes in different molecular pathways, KEGG analysis was conducted using the KEGG orthology-based annotation system (DAVID 6.8 https://david.ncifcrf.gov/). The significance of pathway correlations was indicated by the resultant enrichment score derived from -log10 (p-value).

### In situ hybridization of miR-26b-3p and AKAP2

Cell-climbing sheets were placed in six-well plates and seeded with 2.0 × 105 chondrocytes/well. Upon reaching about 80% confluence, cells were fixed sequentially with 4% paraformaldehyde for 30 min, permeabilized with 0.5% Triton X-100 for 10 min, and blocked with prehybridization solution (1 mL/well) for 30 min. Then, 500 μL of hybridization solution containing the hsa-miR-26b-3p probe labeled with 500 ng/mL FAM and AKAP2 probe labeled with Cy3 was added and incubated at 65 °C for 48 h. The subsequent steps of washing and DAPI staining (miRCURY LNA miRNA ISH kit; Thermo Fisher Scientific, Inc.) were performed per the manufacturer’s instructions. Intracellular colocalization of miR-26b-3p and AKAP2 was assessed using fluorescence microscopy.

### Animal experiments and miR-26b-3p overexpression

six pregnant rats with 50 male cubs were used. The detailed grouping information was shown in Additional file 2: Fig. S1. All animals were given free access to food and water (Beijing Keao Xieli Feed Company), housed at 22–24 °C, and subjected to a 12:12-h light/dark cycle. MiR-26b-3p was overexpressed using plasmids of miR-26b-3p overexpression or chondrocyte exosomes of miR-26b-3p overexpression via tail vein injections on 1-month-old rats. All animal experiments were performed in accordance with the Chinese guidelines for the care and use of laboratory animals and were approved by the animal ethics committee of Nanchang University.

### Tissue sections, bromodeoxyuridine (BrdU), and calcein experiments

After rats were sacrificed, chondrocytes from their femur growth plate were harvested. The bone tissues of the distal femur and proximal one-third of the tibia were fixed using 4% paraformaldehyde overnight at 4 °C. Specimens were decalcified with EDTA solution that was changed regularly for four weeks. Dehydrated specimens were embedded in paraffin and cut into 4-μm-thick sections. Coronal paraffin sections were used for subsequent histochemical staining, in situ hybridization of miR-26b-3p and AKAP2, and BrdU and calcein experiments. BrdU was used to determine chondrocyte proliferation; accordingly, rats were sacrificed 2 h after an intraperitoneal injection of 100 μg/g BrdU. Calcein was used to determine the growth rate of growth plates. For this experiment, rats were sacrificed 72 h after an intraperitoneal injection of 10 μg/g calcein. Coronal paraffin sections were prepared according to the procedures described above. BrdU and calcein were detected using immunohistochemistry. The daily growth rate in rats was calculated based on the farthest distance from the cartilage-bone junction to the calcein-labeled line.

### Immunohistochemistry

The prepared 4-μm-thick paraffin sections were deparaffinized and hydrated sequentially using xylene and ethanol. For antigen retrieval, the sections were heated with sodium citrate buffer in water to boiling for 6 min in a microwave oven. Next, 3% H_2_O_2_ was added and allowed to react for 15 min, followed by the addition of 5% goat serum at room temperature for 60 min to block nonspecific sites. The sections were incubated with the primary antibody (COL10A, RUNX2, OPN, OCN and AKAP2) overnight. The secondary antibody at a 1:200 dilution corresponding to the species was added and incubated for 1 h. The color was developed and photographed. Image-Pro Plus 6.0 (Media Cybernetics, Inc., Rockville, MD, USA) was used for analysis.

### Statistical analysis

In order to analyze clinical parameters and gene expression differences, *t*-tests, ANOVAs, and Tukey's test were used, as well as chi-square tests. We utilized a receiver operating characteristic (ROC) analysis to further investigate the diagnostic efficacy of miR-26b-3p in differentiating the ISS group from normal controls. The ROC curve and area under the ROC curve (AUC) were calculated with MedCalc for Windows, version 19.3.0 (MedCalc Software, Ostend, Belgium). Less than 0.001 (***), 0.01 (**) and 0.05 (*) were considered statistically significant. The statistical analyses were performed using the SPSS V20.0 software (IBM Corp., NY, USA).

## Results

### Analysis of plasma exosomes

**Fig. 1:**
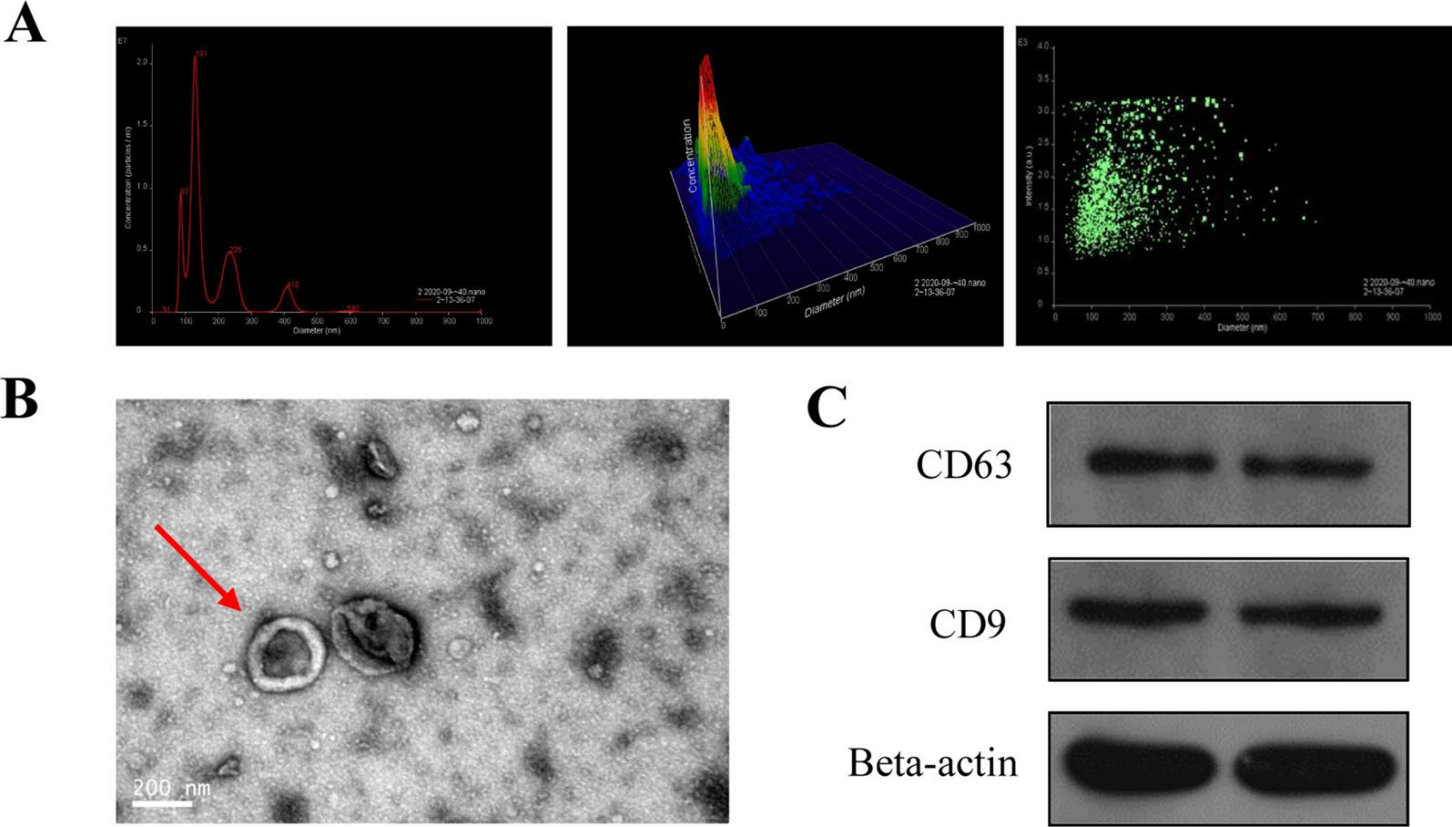
Isolation and identification of plasma exosomes. **A** Nano-Sight analysis verified that the size of extraction is about 100 nm. **B** The morphology of the extraction was further examined via transmission electron microscope. **C** Western blot identified positive expression of exosomes markers, CD63 and CD9, in the extraction samples

Exosomes were isolated from the plasma of patients with ISS and healthy children using ultracentrifugation and identified using transmission electron microscopy (TEM) and western blotting. TEM revealed the isolated exosomes to be cup-shaped or round, with a diameter of approximately 100 nm (Fig. 1A). Nano-Sight analysis revealed most exosomes to be about 100 nm in size (Fig. 1B). Additionally, western blotting suggested that these vesicles positively expressed CD63 and CD9, which are protein markers in exosomes. Hence, these vesicles were confirmed to be exosomes (Fig. 1C).

### Suppression of proliferation and endochondral ossification of human chondrocytes by plasma exosomes isolated from patients with ISS

**Fig. 2:**
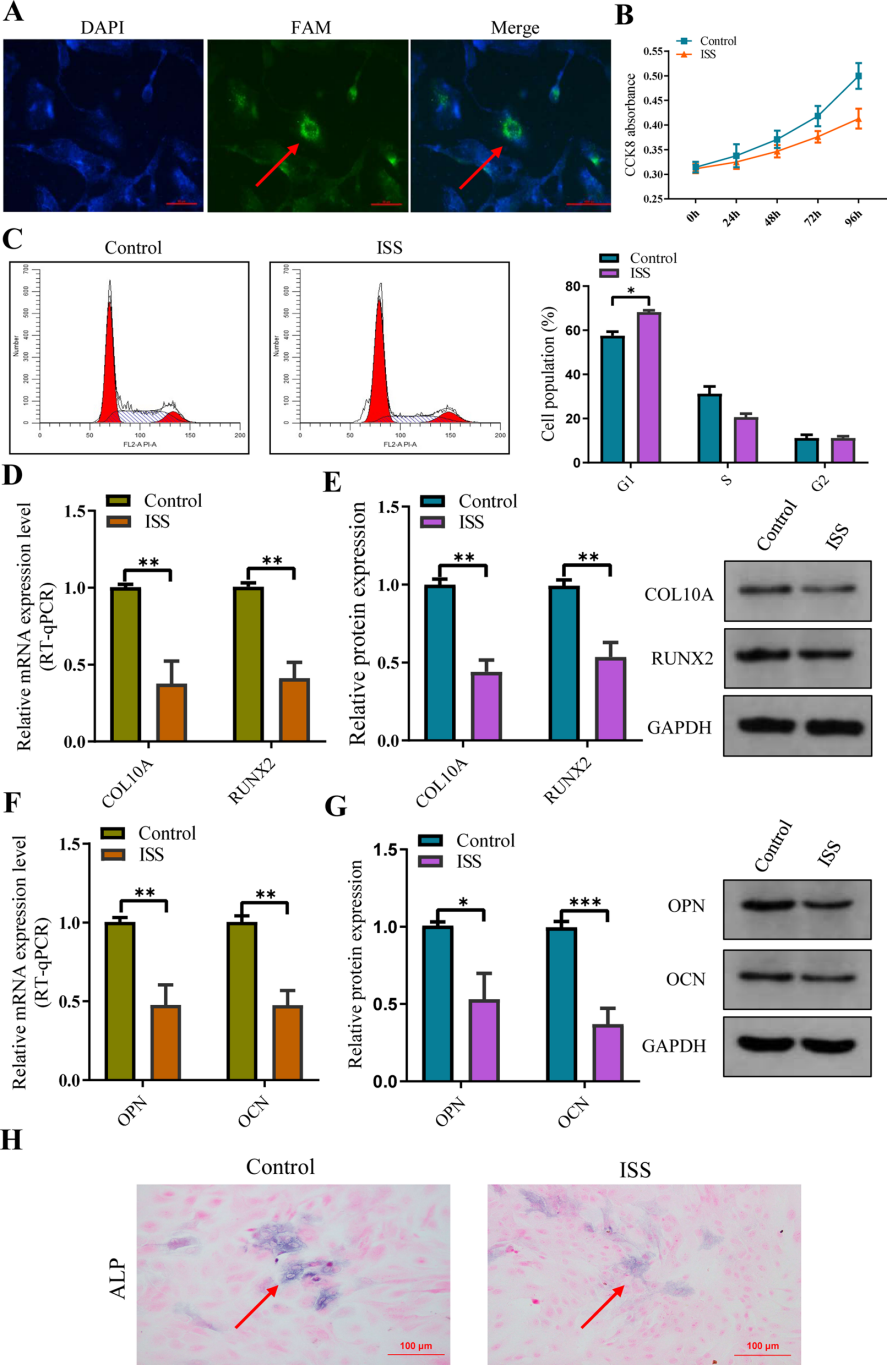
Plasma exosomes of ISS children suppressed the proliferation and bone formation of normal human chondrocytes in vitro. **A** The FAM-labeled exosomes (Green fluorescence) uptake by chondrocytes (Blue fluorescence) was confirmed. **B** CCK8 showed that human chondrocyte proliferation was suppressed after human chondrocytes were cocultured with ISS plasma exosomes. **C** Flow cytometric analysis revealed that cell cycle was arrested in the G0/G1 phase. **D**, **E** Western blot and RT-qPCR identified the marker genes of chondrocyte hypertrophic differentiation, COL10A (collagen type X) and RUNX2, showed obvious downregulation after human chondrocytes were cocultured with ISS plasma exosomes. **F**, **G** Western blot and RT-qPCR observed that the osteogenic genes, OCN and OPN, were also downregulated after human chondrocytes were cocultured with ISS plasma exosomes. **H** The activity of ALP was reduced after co-culture of human chondrocytes with ISS plasma exosomes. The data are presented as the mean ± SD. n = 3. Two groups were compared using *T*-test, *P < 0.05, ^**^P < 0.01, ^***^P < 0.001 vs. control

We examined the effect of plasma exosomes isolated from the ISS and control groups on human chondrocytes. Human chondrocytes were cocultured with ISS's plasma exosomes (Fig. 2A). CCK-8 assay showed that the proliferation rate of human chondrocytes in the ISS group was lower than that in the control group, indicating that exosomes from the ISS group inhibited human chondrocyte proliferation (Fig. 2B). Additionally, flow cytometry findings showed that there were more chondrocytes in the G0/G1 phases in the ISS group and fewer in the S and G2/M phases. These findings illustrated that the cell cycle arrest in exosomes was more significant in the G1 phase in the ISS group than in the control group (Fig. 2C). RT-qPCR and western blotting revealed that exosomes from the ISS group had significantly lower expression levels of marker genes of chondrocyte hypertrophic differentiation (COL10A and RUNX2) and the osteogenic genes (OCN and OPN) than those from the control group (Fig 2D–G). These results demonstrated that ISS exosomes inhibited the endochondral ossification of human chondrocytes. Additionally, ALP activity was determined on samples from both the ISS and control groups (Fig. 2H). The results showed a decrease in ALP activity in samples from the ISS group. In summary, plasma exosomes inhibited the proliferation, hypertrophic differentiation, and endochondral ossification of human chondrocytes obtained from patients with ISS.

### Differential expression of miRNAs in exosomes obtained from the ISS group

**Fig. 3:**
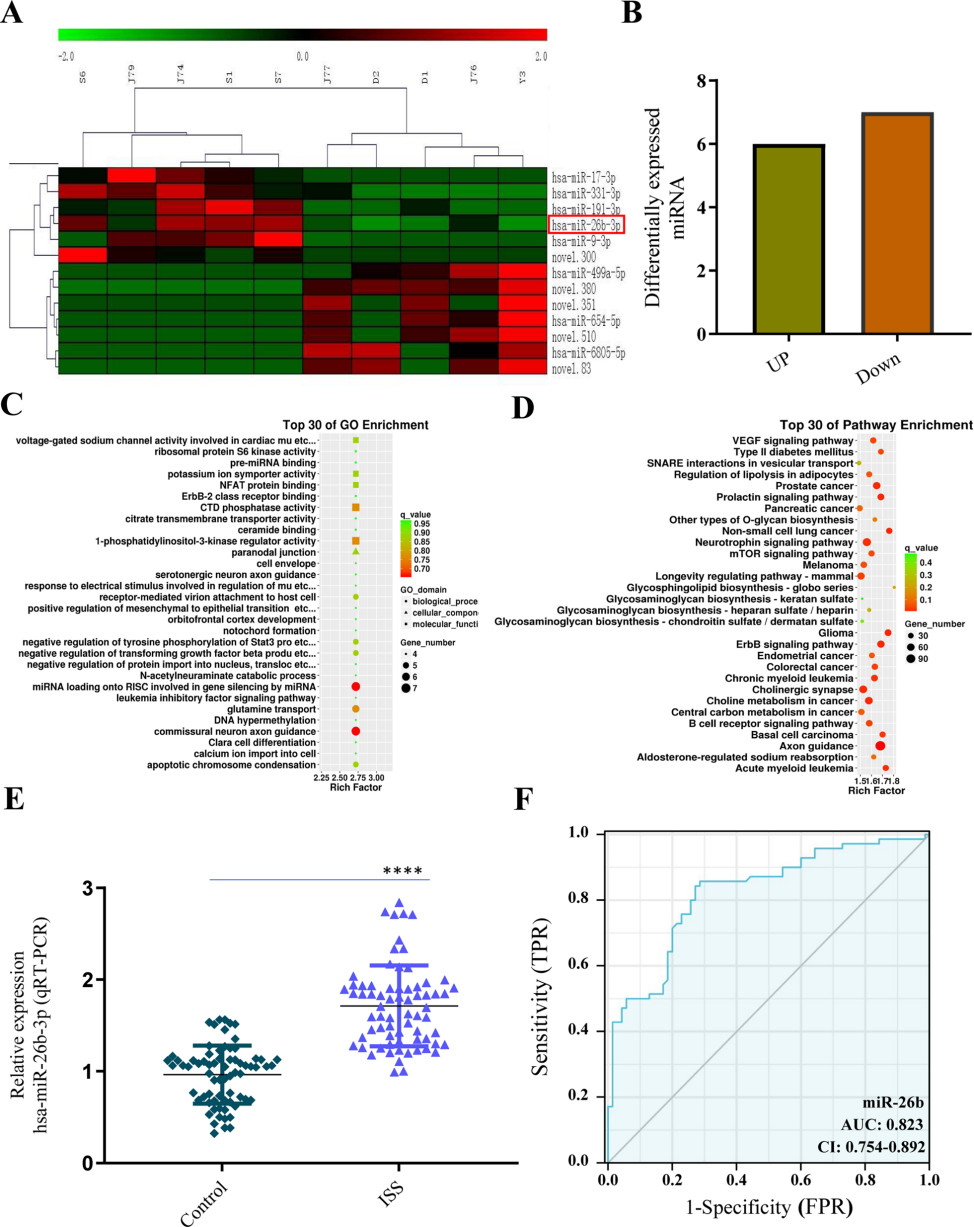
MiR-26b-3p overexpression was identified via miRNA high-throughput sequencing and RT-qPCR, and miR-26b-3p showed high diagnostic efficiency in ISS children. **A**, **B** Thirteen differentially expressed miRNAs were identified, including 6 upregulation and 7 downregulation. **C**, **D** GO and KEGG analyses were employed for further enriching the 13 differentially expressed miRNA. **E** To double-check the veracity, the top three upregulated miRNA s were chosen. The greatest significant difference was detected in miR-26b-3p. **F** An AUC of 0.823 (95% CI, 0.754 – 0.892) was observed. MiR-26B-3P, with the best cutoff point of > 1.173, had a specificity of 71.4% and a sensitivity of 85.7% in discriminating children with ISS from normal control children. The data are presented as the mean ± SD. n = 3. ***P < 0.001 vs. control

Plasma exosomes from five patients with ISS and five matched controls were used for miRNA high-throughput sequencing. The results showed that there were 13 differentially expressed miRNAs in the exosomes from the ISS group, including 6 upregulated miRNAs and 7 downregulated miRNAs (P < 0.05, |logFC| value ≥ 2.0) (Fig. 3A and B). GO and KEGG analyses further enriched these differentially expressed miRNAs. The top 30 KEGG pathways and GO enrichments are shown in Fig. 3C and D. The 3 most upregulated miRNAs were selected for further analysis and miR-26b-3p was found to have the highest fold of upregulation (Fig. 3E). Subsequently, using miR-26b-3p as a diagnostic marker, we analyzed how well it distinguished ISS children from normal children. The present study identified an AUC of 0.823 (95% CI 0.754–0.892). With a cutoff of > 1.173, miR-26b-3p had an 71.4% specificity and 85.7% sensitivity (Fig. 3F).

### Suppression of chondrocytes proliferation and endochondral ossification by miR-26b-3p in exosomes

**Fig. 4:**
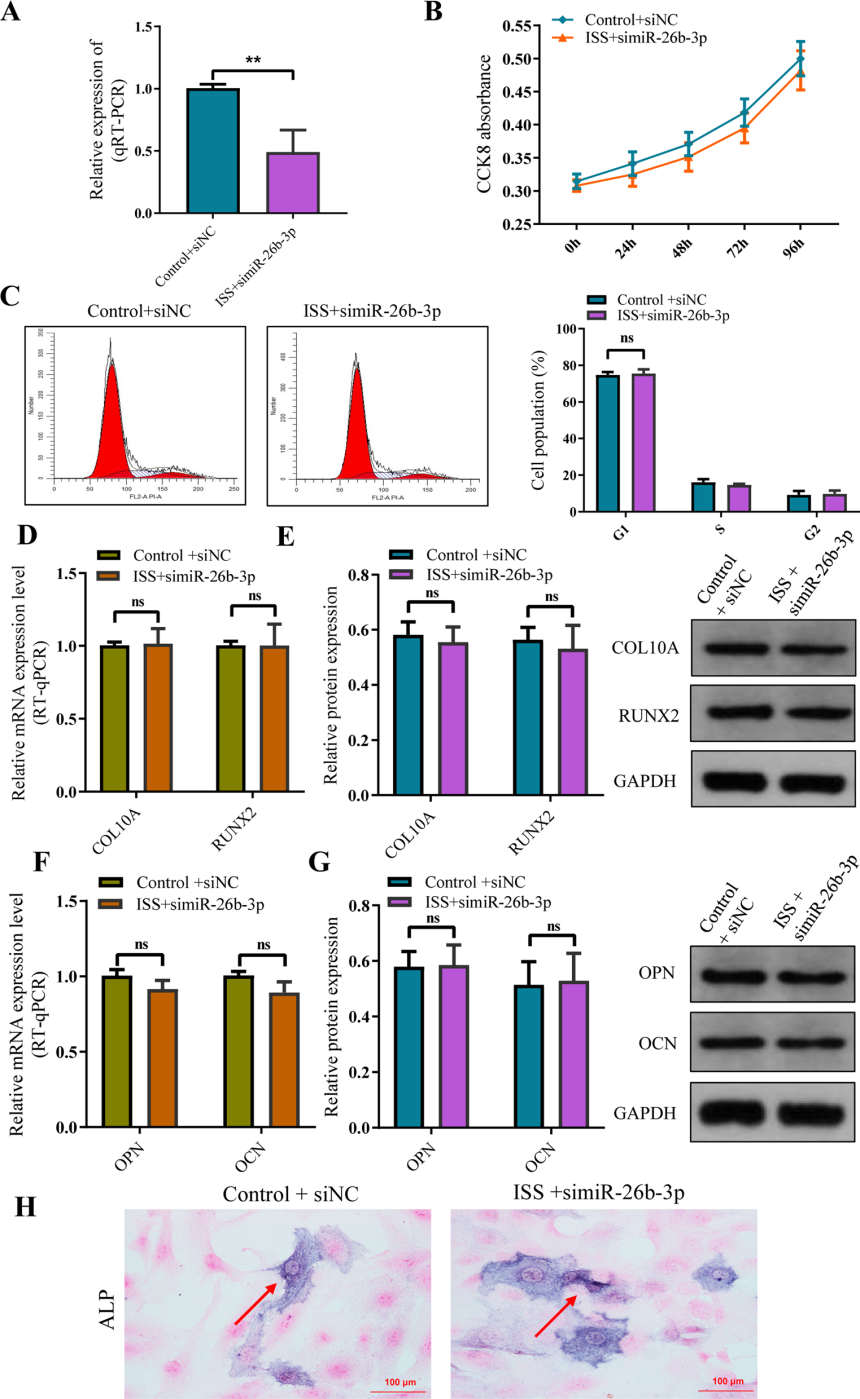
Inhibition of the proliferation and bone formation of normal human chondrocytes caused by ISS exosomes was significantly improved after miR-26b-3p was downregulated. **A** The expression of miR-26b-3p was significantly suppressed by siRNA. **B** CCK8 assay showed a significant improvement in the inhibition of chondrocyte proliferation caused by ISS exosomes after downregulation of miR-26b-3p. **C** Flow cytometric analysis demonstrated an improvement in cell cycle progression after downregulation of miR-26b-3p. **D**–**G** Western blot and RT-qPCR analyses revealed a significant improvement in the expression of chondrocyte hypertrophic differentiation markers (collagen type X and RUNX2) as well as osteogenic genes (OCN and OPN) after downregulation of miR-26b-3p. **H** The activity of ALP was enhanced after downregulation of miR-26b-3p. The data are presented as the mean ± SD. n = 3. Two groups were compared using *T*-test or three groups were compared using ANOVA followed by Tukey’s test. **P < 0.01, ns, not significant vs. control

Human chondrocytes were co-cultured with plasma exosomes of ISS patients or healthy children. miR-26b-3p in ISS plasma exosomes were silenced, which reversed the inhibition of human chondrocytes proliferation, hypertrophic differentiation, and endochondral ossification (Fig. 4A–I).

**Fig. 5:**
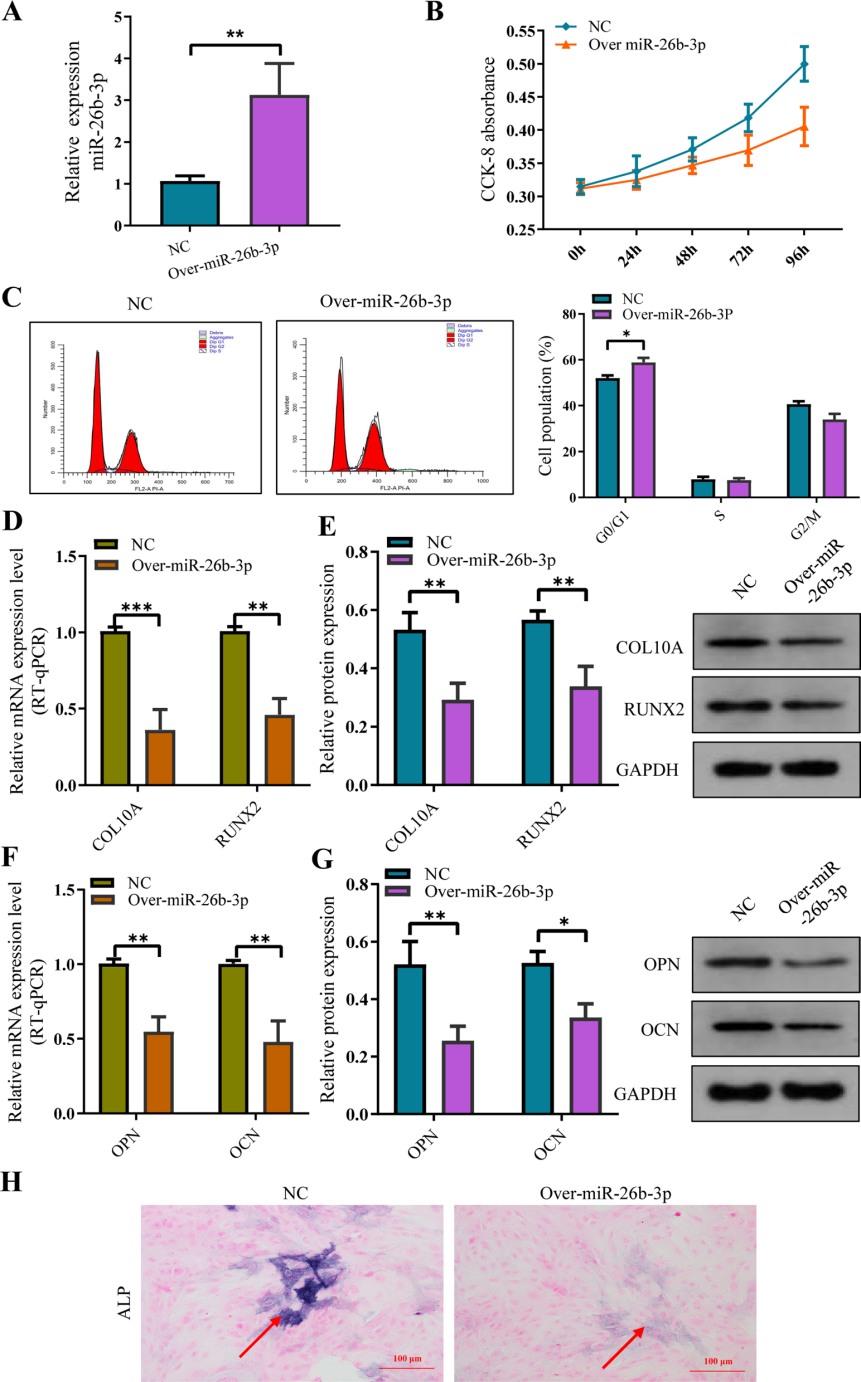
The overexpression of miR-26b-3p suppressed the proliferation and bone formation of normal human chondrocytes. **A** miR-26b-3p was overexpressed in human chondrocytes. **B** CCK8 showed that upregulating miR-26b-3p inhibited proliferation of human chondrocytes**. C** Flow cytometric analysis revealed that cell cycle was arrested in the G0/G1 phase after the overexpression of miR-26b-3p. **D**, **E** Western blot and RT-qPCR identified the marker genes of chondrocyte hypertrophic differentiation, COL10A and RUNX2, showed obvious downregulation after the overexpression of miR-26b-3p. **F**, **G** Western blot and RT-qPCR observed that the osteogenic genes, OCN and OPN, were also downregulated following the overexpression of miR-26b-3p. **H** The activity of ALP was reduced in mineralization after the overexpression of miR-26b-3p. The data are presented as the mean ± SD. n = 3. Two groups were compared using *T*-test. *P < 0.05, **P < 0.01, ***P < 0.001 vs. control

To further investigate the role of miR-26b-3p in chondrocytes, normal human chondrocytes were transfected with pHBLV-miR-26b-3p (Additional file 3: Fig. S2), which upregulated miR-26b-3p expression (Fig. 5A). CCK-8 assay revealed that miR-26b-3p overexpression decreased the growth rate of human chondrocytes (Fig. 5B). Additionally, flow cytometry showed that a higher number of chondrocytes in the miR-26b-3p–upregulated group were in the G0/G1 phases and fewer in the S and G2/M phases. The results illustrated that cell cycle arrest was more significant in the G1 phase in the miR-26b-3p–upregulated group compared with the control group (Fig. 5C). RT-qPCR and western blotting revealed that miR-26b-3p–upregulated group had significantly lower expression levels of chondrocyte hypertrophic differentiation markers (COL10A and RUNX2) and osteogenic genes (OCN and OPN) compared with the control group, indicating that miR-26b-3p upregulation inhibited endochondral ossification of human chondrocytes (Fig. 5D–G). Additionally, both miR-26b-3p–upregulated and the control groups exhibited ALP activity, indicative of a decrease in both ALP activity in the miR-26b-3p–upregulated group (Fig. 5H). The results demonstrated that miR-26b-3p overexpression inhibited proliferation, hypertrophic differentiation, and endochondral ossification of human chondrocytes.

**Fig. 6:**
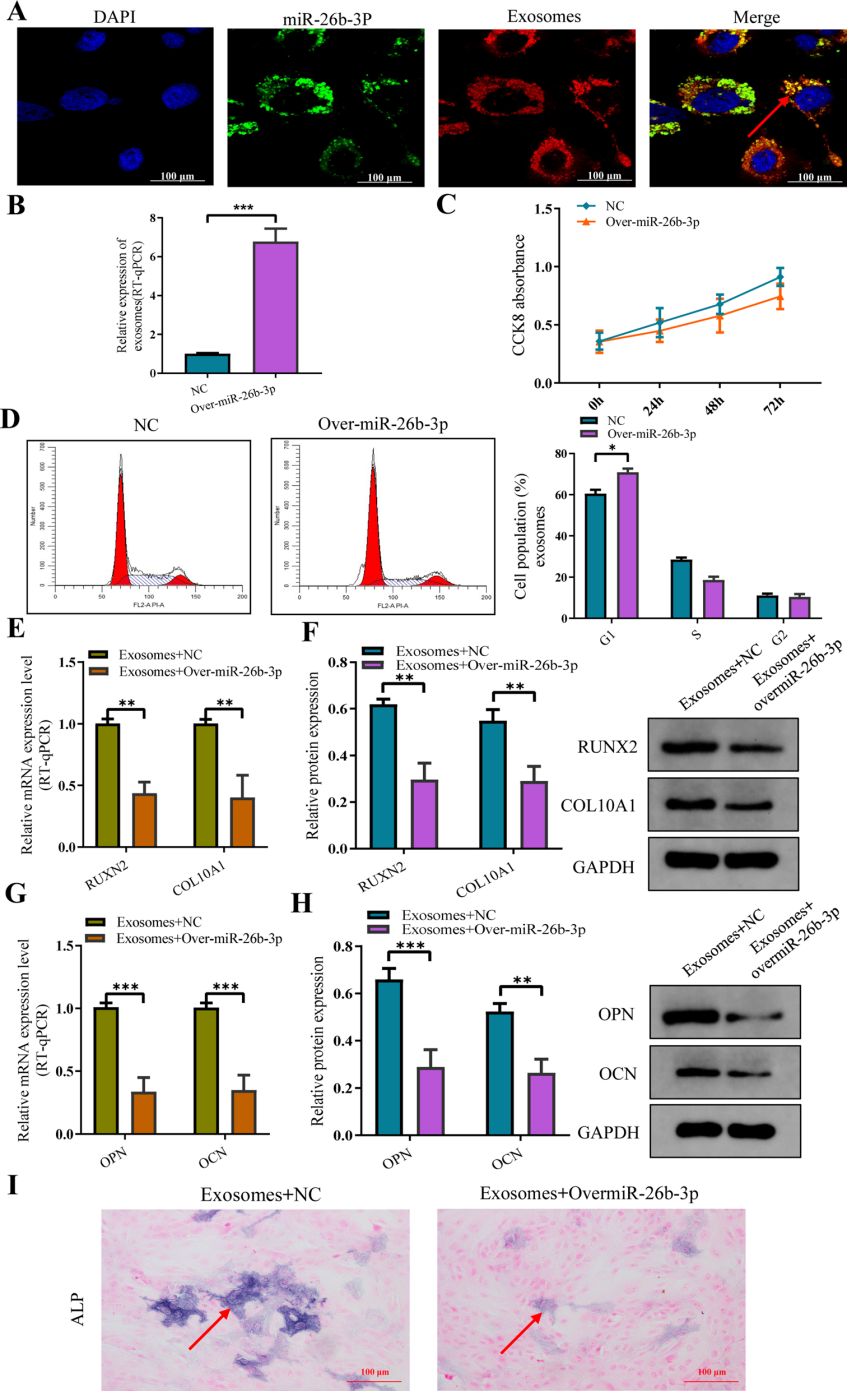
The exosomes with miR-26b-3p overexpression of chondrocyte suppressed the proliferation and bone formation. **A** Internalization of exosomes by chondrocyte examined by laser scanning confocal microscope. **B** miR-26b-3p was overexpressed in chondrocytes. **C** CCK8 showed that upregulating miR-26b-3p inhibited proliferation of chondrocytes after the overexpression of miR-26b-3p. **D** Flow cytometric analysis revealed that cell cycle was arrested in the G0/G1 phase after the overexpression of miR-26b-3p. **E**, **F** Western blot and RT-qPCR identified the marker genes of chondrocyte hypertrophic differentiation, COL10A and RUNX2, showed obvious downregulation after the overexpression of miR-26b-3p. **G**, **H** Western blot and RT-qPCR observed that the osteogenic genes, OCN and OPN, were also downregulated after the overexpression of miR-26b-3p. **I** The activity of ALP was reduced in mineralization after the overexpression of miR-26b-3p. The data are presented as the mean ± SD. n = 3. Two groups were compared using *T*-test. *P < 0.05, **P < 0.01, ***P < 0.001 vs. control

The chondrocyte exosomes with miR-26b-3p overexpression were successfully constructed according to the manufacturer's instructions. Internalization of exosomes by chondrocyte examined by laser scanning confocal microscope (Fig. 6A). Subsequently, the chondrocyte exosomes with miR-26b-3p overexpression were cocultured with human chondrocytes in order to further verify the role of exosomes miR-26b-3p overexpression on proliferation, hypertrophic differentiation, and endochondral ossification of human chondrocytes. As expected, the proliferation, hypertrophic differentiation, and endochondral ossification in human chondrocytes were suppressed again (Fig. 6B–I).

### miR-26b-3p in exosomes inhibits proliferation and endochondral ossification of chondrocytes via the AKAP2 in vitro

**Fig. 7:**
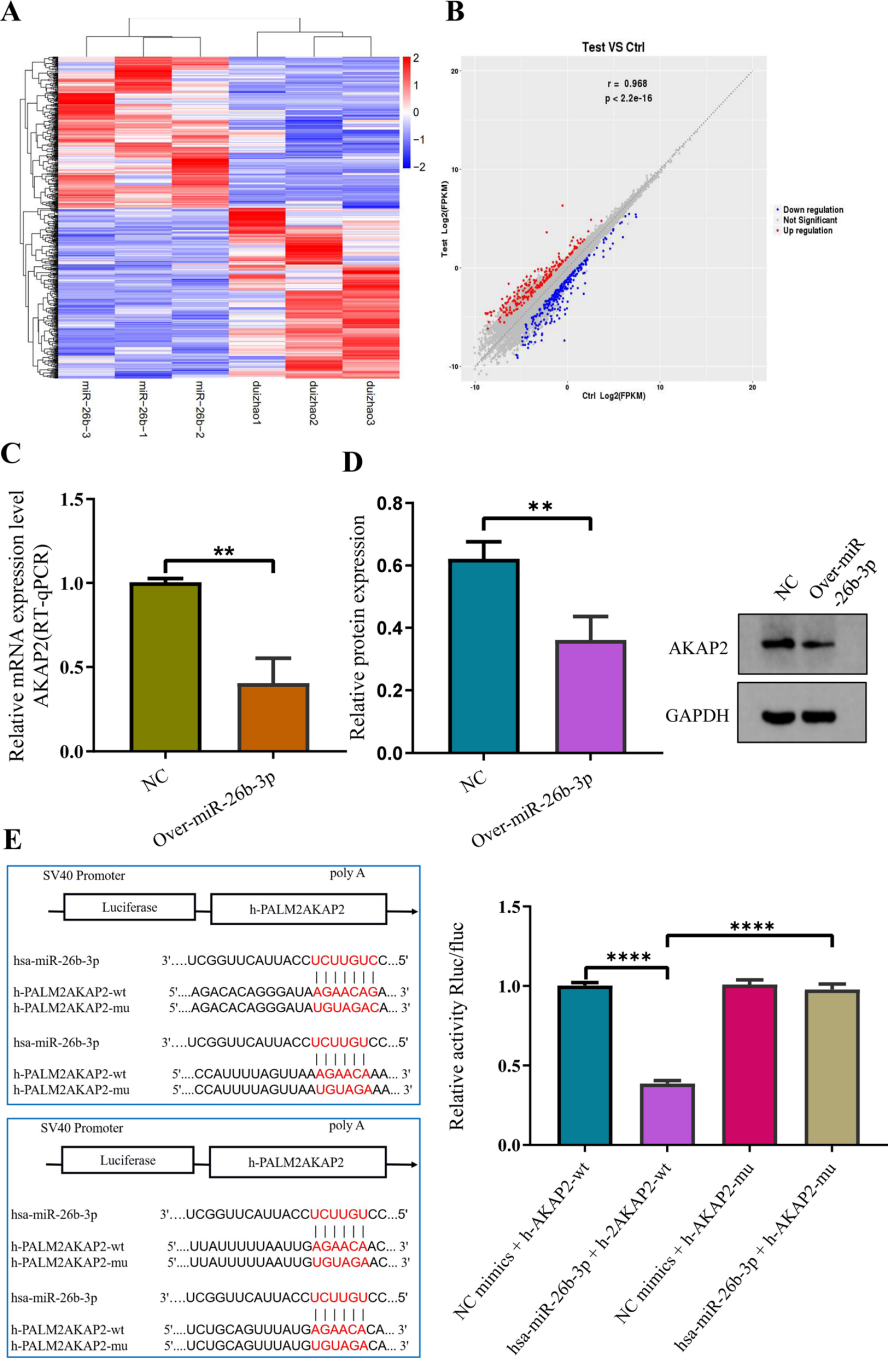
miR-26b-3p in exosomes inhibits chondrocytes proliferation and endochondral ossification via the AKAP2 in vitro*.* **A** mRNA microarray assays was performed to identify differentially expressed genes after overexpression of miR-26b-3p. **B** The results showed that 316 mRNAs were differentially expressed, with 126 upregulated and 190 downregulated mRNAs. **C**, **D** Western blot and RT-qPCR analyses confirmed that AKAP2 expression was decreased upon overexpression of miR-26b-3p. **E** Luciferase activity assays revealed that co-transfection of AKAP2 with its mutant fragment restored luciferase activity, indicating that AKAP2 is a direct target of miR-26b-3p. The data are presented as the mean ± SD. n = 3. Two groups were compared using *T*-test. **P < 0.01, ***P < 0.001 vs. control

To further determine the downstream target genes of miR-26b-3p, differentially expressed mRNAs were analyzed using mRNA high-throughput sequencing after upregulation of miR-26b-3p. The results showed that there were 316 differentially expressed mRNAs, including 126 upregulated mRNAs and 190 downregulated mRNAs (P < 0.05, |logFC|–value > 2.0) (Fig. 7A, B). Then, the downstream mRNA of miR-26b-3p was predicted using TargetScan and miRDB databases. The 7 most mRNAs were selected for further analysis (P62, ATG5, MTOR, NIN, MIBI, SERPINA1 and AKAP2) (Additional file 4: Fig. S3). After intersection, AKAP2 was selected as the downstream candidate gene of miR-26b-3p. Results from RT-qPCR and western blotting indicated that AKAP2 expression was downregulated upon miR-26b-3p overexpression (Fig. 7C, D).

**Fig. 8:**
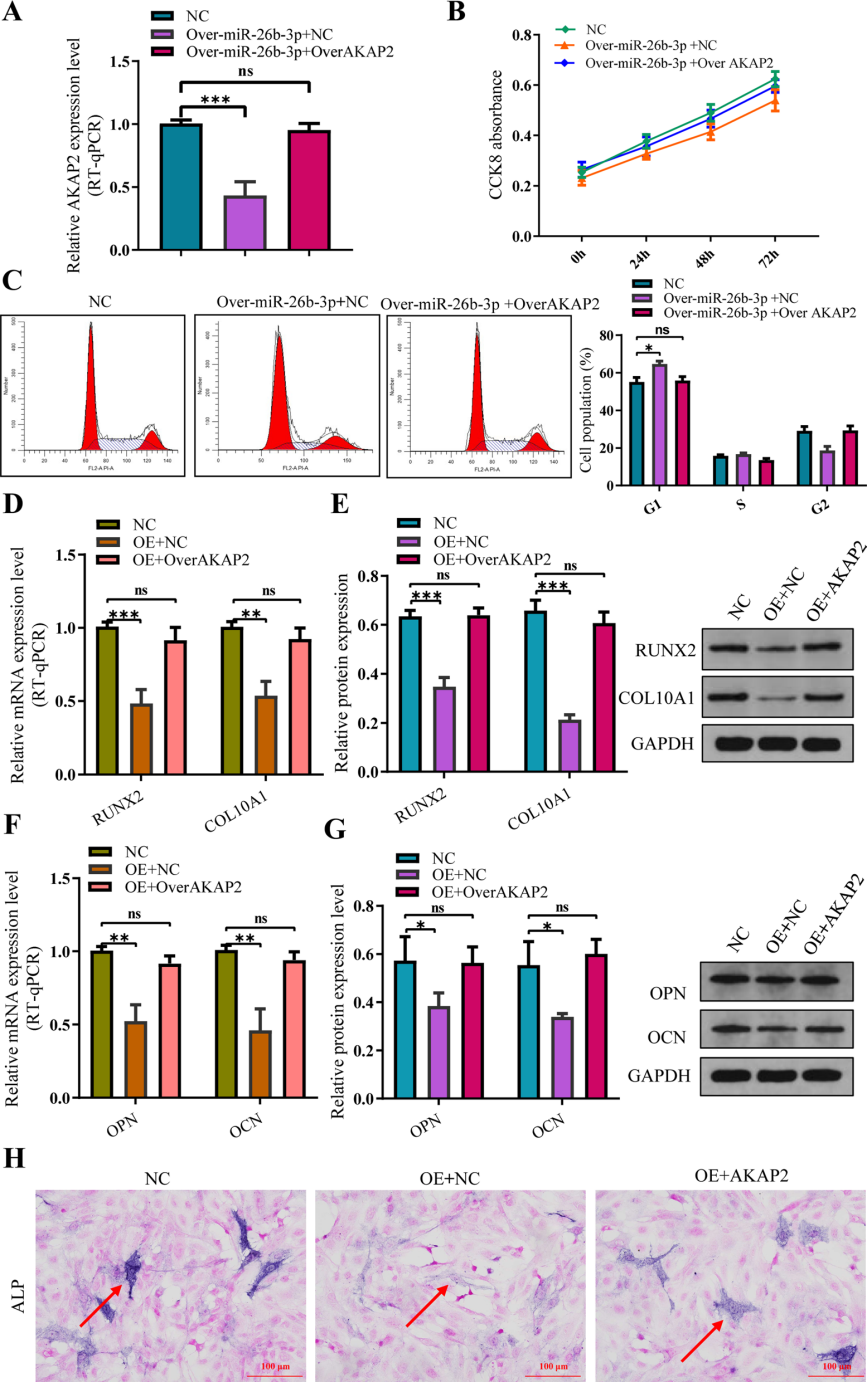
Rescue experiments indicates that AKAP2 overexpression reversed the inhibition of human chondrocyte proliferation caused by overmiR-26b-3P. **A** AKAP2 presented downregulation after overexpression of miR-26b-3p, however, it was significantly improved in the group of miR-26b-3p upregulation + AKAP2. **B**, **C** Although CCK8 and cell cycle were inhibited in the miR-26b-3p mimic + NC group, whereas it was enhanced in the miR-26b-3p mimic + AKAP2 group. **D–G** Compared with the group of miR-26b-3p upregulation, the expression of marker genes of chondrocyte hypertrophic differentiation (COL10A and RUNX2) and the osteogenic genes (OCN and OPN) was also significantly improved in the group of miR-26b-3p upregulation + AKAP2 upregulation. **H** Although the activity of ALP in chondrocytes were suppressed after miR-26b-3p upregulation, it was reversed after AKAP2 was upregulated. The data are presented as the mean ± SD. n = 3. Two groups were compared using *T*-test or three groups were compared using ANOVA followed by Tukey’s test. ns, not significant, *P < 0.05, **P < 0.01, ***P < 0.001 vs. control

Double-luciferase reporter assay revealed that the relative luciferase activity in miR-26b-3p + AKAP2 3′UTR—wildtype was significantly inhibited, whereas that in miR-26b-3p + AKAP2 3′UTR—mut increased. Results of the luciferase assay confirmed that miR-26b-3p could target AKAP2 regulation (Fig. 7E). RT-qPCR revealed that the expression levels of AKAP2 were inhibited in the miR-26b-3p mimic + NC group, whereas it was enhanced in the miR-26b-3p mimic + Over-AKAP2 group (Fig. 8A). Rescue experiments revealed that though CCK8 and cell cycle were inhibited in the miR-26b-3p mimic + NC group, whereas it was enhanced in the miR-26b-3p mimic + Over-AKAP2 group (Fig. 8B, C). In the miR-26b-3p mimic + Over-AKAP2 group, inhibition of the growth and proliferation of human chondrocytes was reversed. Besides, the protein expression levels of OCN, OPN, RUNX2, and COL10A were also increased (Fig. 8D–G). The ALP activity improved as determined. (Fig. 8H). In vitro experiments revealed that upregulation of miR-26b-3p inhibits the chondrocytes proliferation, hypertrophic differentiation, and endochondral ossification via the AKAP2 axis.

### MiR-26b-3p inhibits estrogen receptor α (ER-α) via AKAP2/ERK1/2 axis in human chondrocytes.

**Fig. 9:**
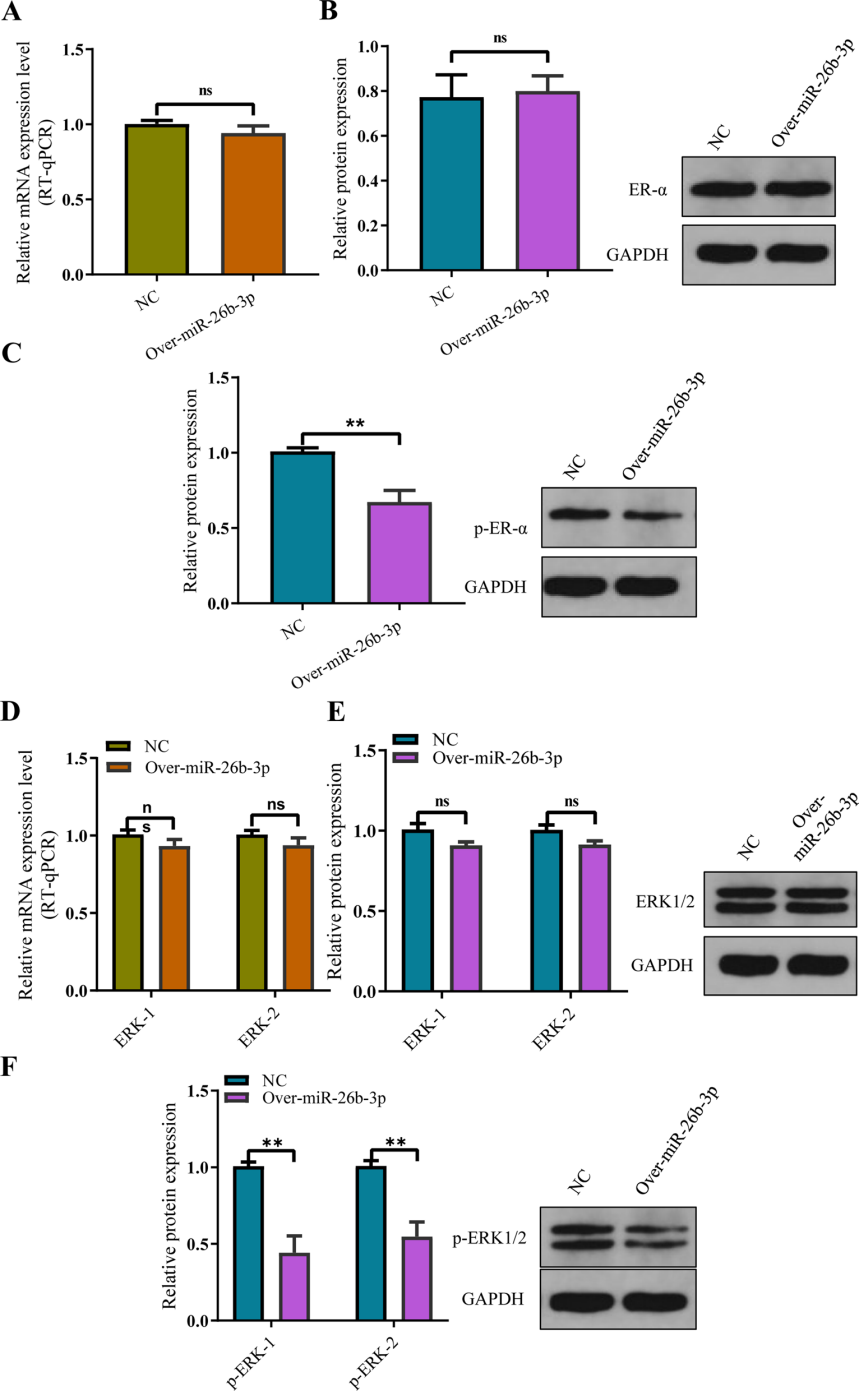
MiR-26b-3p inhibits estrogen receptor α (ER-α) via AKAP2/ERK1/2 axis in human chondrocytes. **A**, **B** The relative mRNA and protein expression levels of ER-α did not show statistical difference between the group of miR-26b-3p overexpression and control group. **C** The expression of p-ER-α was obviously downregulated in the group of miR-26b-3p overexpression compared with control group. **D**, **E** The expression of ERK1/2 protein and p-ERK1/2 protein. Similarly, significant differences on the relative mRNA and protein expression of ERK1/2 were not observed between the group of miR-26b-3p overexpression and control group in our study. **F** The expression of p-ERK1/2 protein was significantly decreased in the group of miR-26b-3p overexpression compared with control group. The data are presented as the mean ± SD. n = 3. Two groups were compared using *T*-test or three groups were compared using ANOVA followed by Tukey’s test. ns, not significant, **P < 0.01, ***P < 0.001 vs. control

Lin et al. [32] observed that miR-26b-3p upregulation inhibited osteoblast differentiation via direct targeting ER-α, which was determined by dual luciferase assay. However, we found the relative mRNA and protein expression levels of ER-α did not show statistical difference between the group of miR-26b-3p overexpression and control group (Fig. 9A, B). This indicates that unlike pre-osteoblast cell, miR-26b-3p cannot directly regulate the ER-α in human chondrocytes. It is an interesting finding that though the significant difference on the relative mRNA and protein expression levels of ER-α was not observed between the group of miR-26b-3p overexpression and control group, we found the expression of p-ER-α was obviously downregulated in the group of miR-26b-3p overexpression compared with control group (Fig. 9C).

Wang et al. [33] reported that AKAP2 downregulation can suppress the function of ERK1/2 protein via decreasing p-ERK1/2 activity, not decreasing the expression of ERK1/2 protein. Subsequently, we also detected the expression of ERK1/2 protein and p-ERK1/2 protein. Similarly, significant differences on the relative mRNA and protein expression of ERK1/2 were not observed between the group of miR-26b-3p overexpression and control group in our study (Fig. 9D, E). However, the expression of p-ERK1/2 protein was significantly decreased in the group of miR-26b-3p overexpression compared with control group (Fig. 9F). Previous study [34] have confirmed that decreasing p-ERK1/2 activity can inhibit the levels of p-ER-α. In the present study, the authors observed that though there are not significant difference on the relative mRNA and protein expression levels of ER-α between the group of miR-26b-3p overexpression and control group, the levels of p-ER-α were obviously downregulated in the group of miR-26b-3p overexpression compared with control group. Moreover, decreasing p-ERK1/2 activity was also shown in the group of miR-26b-3p overexpression. This indicates that miR-26b-3p suppresses the function of ER-α via inhibiting the ER-α phosphorylation, not the relative mRNA and protein expression of ER-α in human chondrocytes.

### miR-26b-3p in exosomes can inhibit proliferation and endochondral ossification of growth plate chondrocytes via the AKAP2 axis in vivo, leading to ISS

**Fig. 10:**
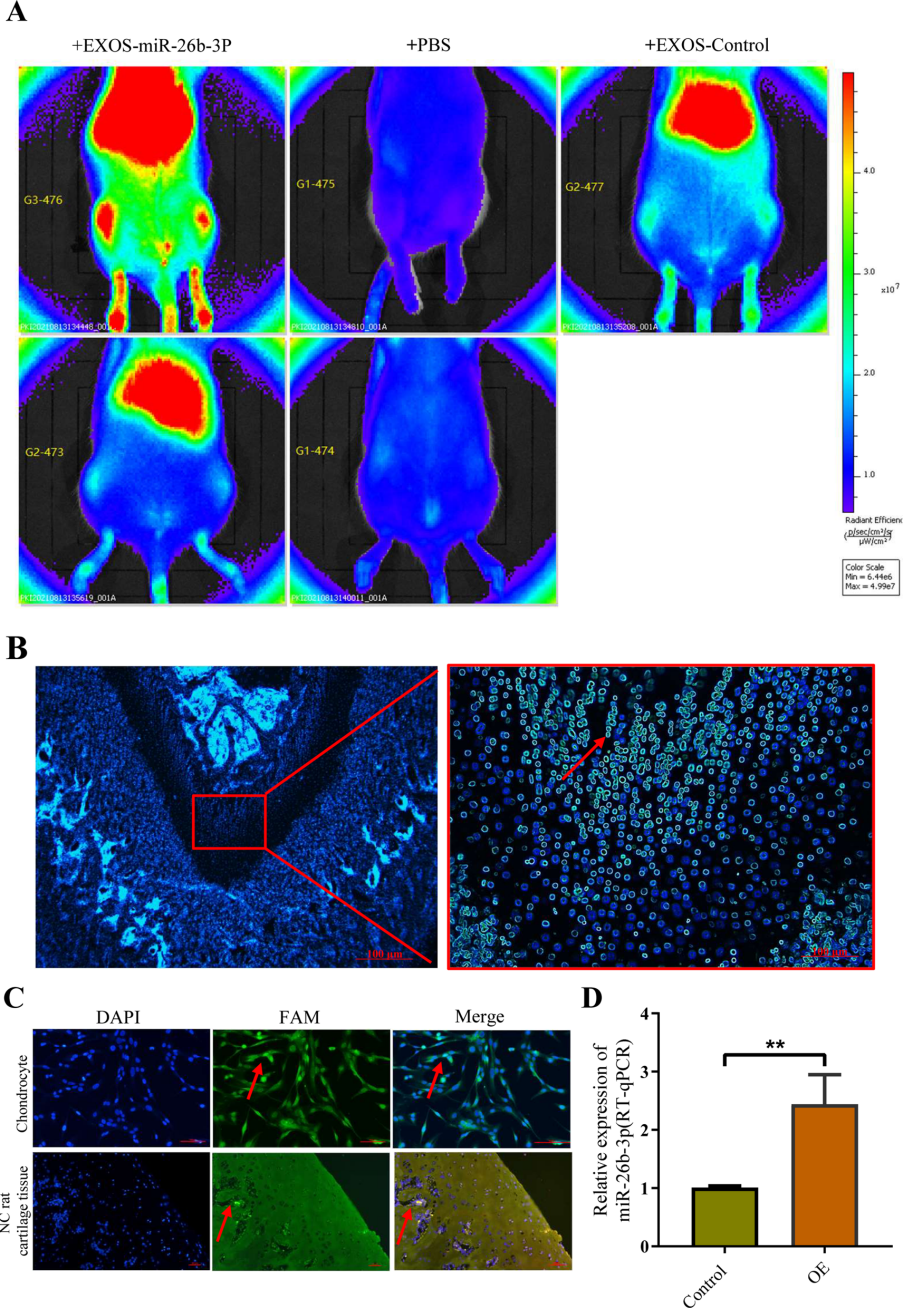
An experiment to determine overexpression was performed in vivo. **A** An experiment to determine overexpression was performed, where in SD rats were administered exosomes containing overexpressed miR-26b-3p via a tail vein injection. Intravital imaging revealed that fluorescence-labeled exosomes were highly concentrated in both lower limbs after 48 h of injection. Exosomes were labelled with miR-26b-3p (green) and nuclei were stained with DAPI (blue). **B** Immunofluorescence findings from tissue sections confirmed that exosomes and miR-26b-3p entered the growth plate chondrocytes. **C** In situ hybridization confirmed that miR-26b-3p was expressed in the proliferative regions of femoral growth plates in rats**. D** RT-qPCR analysis revealed that miR-26b-3p was highly expressed in growth plate chondrocytes. The data are presented as the mean ± SD. n = 3. Two groups were compared using *T*-test or three groups were compared using ANOVA followed by Tukey’s test. **P < 0.01vs. control

**Fig. 11:**
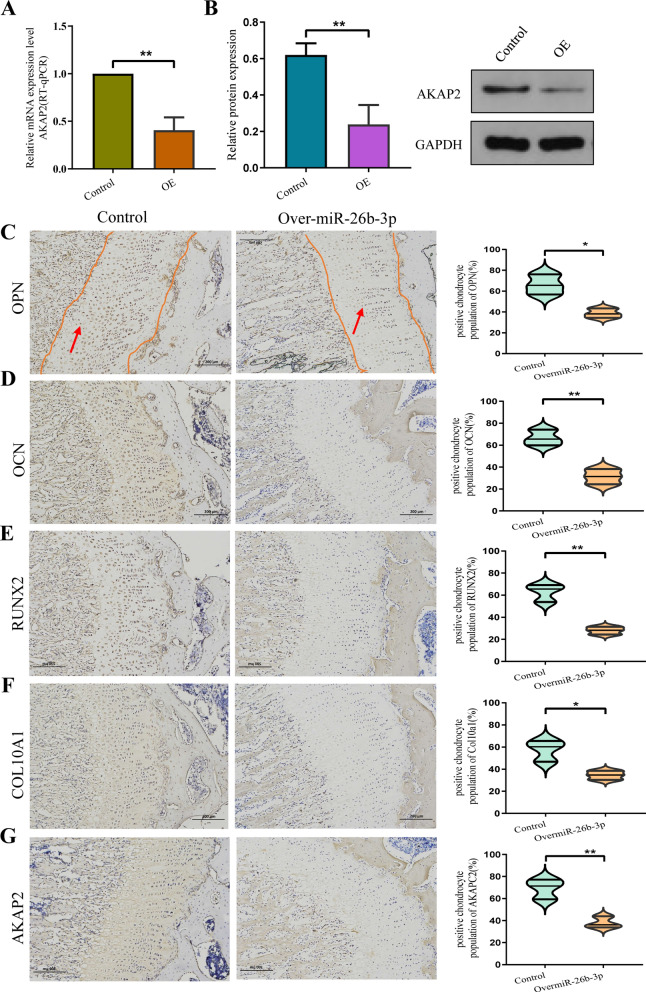
miR-26b-3p in exosomes can inhibit proliferation and endochondral ossification of growth plate chondrocytes via the AKAP2 axis in vivo. **A**–**G** The findings from western blotting, RT-qPCR, and immunohistochemistry revealed that AKAP2, which is the downstream target gene of miR-26b-3p, was significantly downregulated in growth plates. The data are presented as the mean ± SD. n = 3. Two groups were compared using *T*-test or three groups were compared using ANOVA followed by Tukey’s test. *P < 0.05, **P < 0.01, vs. control

**Fig. 12:**
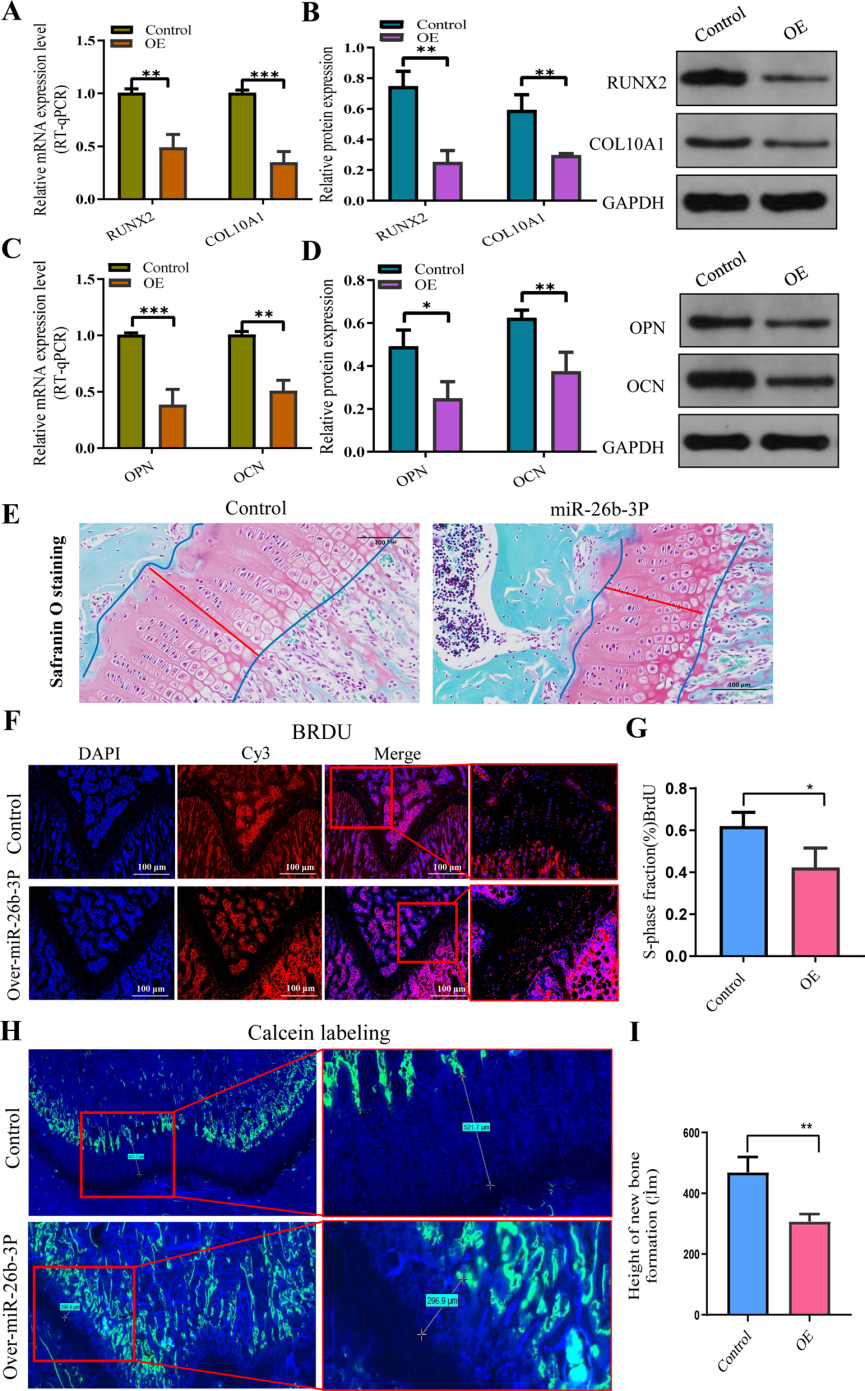
Overexpression of miR-26b-3p suppressed the endochondral ossification of femur growth plate in the rats. **A**–**D** RUNX2, type X collagen, AKAP2, OCN, and OPN, the hallmark genes of differentiation and endochondral ossification, were downregulated. **E** Safranin O-fast green staining showed that the height of the femoral growth plates in the miR-26b-3p–overexpressed group was lesser than that in the NC group. **F**, **G** BrdU labeling demonstrated that the positive markers of the femoral growth plate proliferating regions in the miR-26b-3p–overexpressed group were lower than those in the NC group. **H**, **I** Calcein experiment confirmed that the new osteogenesis rate of growth plate chondrocytes in the miR-26b-3p–overexpressed group decreased significantly. The data are presented as the mean ± SD. n = 3. Two groups were compared using *T*-test. *P < 0.05, **P < 0.01, ***P < 0.001 vs. control

An experiment to determine overexpression was performed, wherein SD rats were administered exosomes containing overexpressed miR-26b-3p via a tail vein injection. Intravital imaging revealed that fluorescence-labeled exosomes were highly concentrated in both lower limbs after 48 h of injection (Fig. 10A). Moreover, immunofluorescence findings from tissue sections confirmed that exosomes and miR-26b-3p entered the growth plate chondrocytes (Fig. 10B). In situ hybridization confirmed that miR-26b-3p was expressed in the proliferative regions of femoral growth plates in rats (Fig. 10C). RT-qPCR analysis revealed that miR-26b-3p was highly expressed in plasma and growth plate chondrocytes (Fig. 10D), whereas findings from western blotting, RT-qPCR, and immunohistochemistry revealed that AKAP2, which is the downstream target gene of miR-26b-3p, was highly expressed in growth plates (Fig. 11A, B, G). Meanwhile, RUNX2, COL10A, OCN, and OPN, the hallmark genes of differentiation and endochondral ossification, were downregulated (Figu. 11C–F, 12A–D). Safranin O-fast green staining showed that the height of the femoral growth plates in the miR-26b-3p–overexpressed group was lesser than that in the NC group (Fig. 12E). BrdU labeling demonstrated that the positive markers of the femoral growth plate proliferating regions in the miR-26b-3p–overexpressed group were lower than those in the NC group (Fig. 12F, G). Calcein experiment confirmed that the new osteogenesis rate of growth plate chondrocytes in the miR-26b-3p–overexpressed group decreased significantly (Fig. 12H, I). After 2 months of miR-26b-3p overexpression in exosomes, the femur, tibia, and overall body height of rats in the experimental group were significantly lower than those in the control group (Additional file 5: Fig. S4A–D). Results from in vivo experiments suggested that miR-26b-3p in exosomes could inhibit proliferation and endochondral ossification of growth plate chondrocytes via the AKAP2 axis, leading to ISS.

To further confirm the effect of miR-26b-3p overexpression on cartilage growth and development in vivo, we constructed adenoviral vectors for miR-26b-3p overexpression and studied the overexpression in vivo after a tail vein injection. Similar to the predicted results, miR-26b-3p overexpression led to the downregulation of AKAP2 expression, resulting in inhibited proliferation and endochondral ossification of growth plate chondrocytes. As a result, the rats showed a short stature phenotype.

## Discussion

Recent studies [27, 28, 35–38] have shown that plasma exosome miRNAs can serve as early diagnostic markers and targeted therapeutic tools in disease management, thereby exhibiting broad application prospects. For example, Peng et al*.* [22] observed that patients with advanced non-small cell lung cancer (NSCLC) had unique plasma exosome miRNA spectra. Hsa-miR-320d, hsa-miR-320c, and hsa-miR-320b were identified as potential biomarkers for predicting the efficacy of immunotherapy in advanced NSCLC. Nie et al. [23] demonstrated that miRNA-231 in exosomes can inhibit metastasis of lung cancer. Jin et al*.* [35] found that overexpression of miRNA-26a-5p in bone marrow mesenchymal stem cell exosomes can mitigate cartilage damage in arthritis. Liu et al. [36] observed that hydrogel-loaded exosomes derived from BMSCs could be anchored in the defect in the cartilage and promote repair. Liu et al*.* [37] demonstrated that exosome miRNA-106b derived from synovial fibroblasts inhibited the proliferation and migration of chondrocytes in rheumatoid arthritis by downregulating PDK4. Zhu et al. [39] reported that exosomal miRNAs derived from osteoblasts and osteoblast precursors, including miR-30d-5p, miR-133b-3p, miR-140-3p, miR-199b, miR-221, and miR-218, play a role in the regulation of osteogenic differentiation. Won et al. [38] detected that high expression of miRNA-26 in exosomes promoted the differentiation of BMSCs into chondrocytes and, also enhanced cartilage repair and regeneration. However, to the best of our knowledge, the role of exosomes in the pathogenesis of ISS has not been reported.

For evaluating the role of exosomes in ISS pathogenesis, the plasma exosomes of ISS children were co-cultured with normal human chondrocytes. Fluorescence labeling revealed the entry of exosomes into chondrocytes. We first observed that plasma exosomes of ISS children suppressed normal chondrocyte proliferation and cell cycle arrested in the G1 phase. Meanwhile, the expression of type X collagen and RUNX2 was downregulated in the ISS group. This indicated that hypertrophic differentiation of chondrocytes was also inhibited. In addition to that, the osteogenic genes *OPN* and *OCN* were decreased, and ALP revealed impairment of endochondral ossification. Our study confirmed that ISS plasma exosomes suppressed human chondrocytes proliferation, hypertrophic differentiation and endochondral ossification. Exosomes contain miRNA, LncRNA, CircRNA, and proteins. Bioinformatics studies have predicted that a third of all human mRNAs are regulated by miRNAs [40]. miRNAs can regulate the proliferation, differentiation, ossification, and metabolism of cartilage and bone cells. Abnormal expression is closely related to orthopedic diseases such as growth plate developmental disorders and osteoarthritis. Therefore, differential expression analysis of miRNAs in plasma exosomes between the ISS children and normal control children was performed by us using high-throughput sequencing. A total of 1177 miRNAs were identified in exosomes, of which 13 were differentially expressed miRNAs with P < 0.05 and log value ≥ 2.0. miRNAs with differential expression fold that were in the top three (hsa-miR-26b-3p, hsa-miR-6805-5p, and hsa-miR-9-3p) were validated using qPCR. Our results verified that miR-26b-3p was highly expressed in the exosomes from the ISS group.

To further analyze that upregulated miR-26b-3p in ISS plasma exosomes is the cause of impairment of chondrocyte proliferation and endochondral ossification, or result, the expression of miR-26b-3p was downregulated after ISS exosome were cocultured with normal human chondrocytes. The rescue experiment showed that downregulation of miR-26b-3p obviously improved the repression of chondrocyte proliferation and endochondral ossification caused by ISS exosomes. Meanwhile, we also upregulated miR-26b-3p expression in human chondrocytes via the plasmid of miR-26b-3p overexpression. As expected, chondrocyte proliferation and endochondral ossification were inhibited once again. This suggests that the upregulated miR-26b-3p in ISS plasma exosomes is responsible for the repression of chondrocyte proliferation and endochondral ossification.

Wang et al. [41] reported that upregulated miR-26b-3p can significantly suppress the proliferation of human umbilical cord-derived mesenchymal stem cells via regulating estrogen receptor in vitro. Geng et al. [42] observed upregulated miR-26b-3p inhibited cell migration and proliferation of glioma. Subsequent study confirmed anthrax toxin receptor 1 is the downstream molecule of miR-26b-3p. Lin et al. [32] demonstrated overexpression of miR-26b-3p can target the estrogen receptor, which can inhibit osteoblast differentiation and block the proliferation of umbilical cord mesenchymal stem cells. However, to date, it is unclear whether miR-26b-3p is expressed in chondrocytes. Meanwhile, the molecular biological mechanisms that miR-26b-3p inhibits the chondrocytes proliferation, hypertrophic differentiation as well as endochondral ossification was also not addressed.

The authors confirmed the expression of miR-26b-3p on human chondrocytes and femoral growth plate cartilage of rat via in situ hybridization. To further identify the downstream target genes of miR-26b-3, mRNA expression microarray was used after miR-26b-3p overexpression, and 316 meaningful differentially expressed mRNAs were identified. Subsequently, mRNAs with the top seven differential expression fold were verified using qPCR. The A-kinase anchoring protein 2 (AKAP2) showed the largest differential expression. Panza et al*.* [43] reported the molecular genetic characteristics of a patient with Kallmann syndrome and bone dysplasia. The patient had gene translocation t(7:9) (p14.1; q31.3), which destroyed the AKAP2 gene on chromosome 9. In situ hybridization revealed that AKAP2 genes are highly expressed in mouse embryonic cartilage. Li et al*.* [44] found a new mutation of the AKAP2 gene, c.2645a > C (p.e882a), in patients with idiopathic scoliosis. To clarify the role of AKAP2 in the growth and development of chondrocytes, AKAP2 was overexpressed in growth plate chondrocytes in vitro experiments [33]. Subsequently, the proliferation, differentiation, and matrix secretion of chondrocytes increased. After AKAP2 silencing, proliferation, differentiation, and matrix secretion of chondrocytes were inhibited. Considering that AKAP2 showed the largest differential expression after the overexpression of miR-26b-3p and that AKAP2 played a key role in the regulation of growth plate cartilage development, we listed AKAP2 was a candidate gene for further study.

In situ hybridization revealed that miR-26b-3p and AKAP2 were co-expressed in human chondrocytes, whereas luciferase expression experiments confirmed that miR-26b-3p combined with AKAP2. More importantly, rescue experiments showed that AKAP2 overexpression could inhibit proliferation, hypertrophic differentiation, and endochondral ossification of chondrocytes induced by miR-26b-3p overexpression. In vitro findings revealed that miR-26b-3p could mediate AKAP2 to inhibit chondrocyte proliferation and endochondral ossification. Wang et al. [33] reported that AKAP2 downregulation can suppress the function of ERK1/2 via decreasing p-ERK1/2 activity, not decreasing the relative mRNA and protein expression of ERK1/2. Given the important regulatory roles of ERK1/2 on chondrocyte proliferation and endochondral ossification, we also detected the expression of ERK1/2. Similarly, significant differences on the relative mRNA and protein expression of ERK1/2 were not observed between the group of miR-26b-3p overexpression and control group in our study. However, the expression of p-ERK1/2 protein was significantly decreased in the group of miR-26b-3p overexpression compared with control group. This suggests that miR-26b-3p inhibits chondrocyte proliferation, hypertrophic differentiation, and endochondral ossification via AKAP2/ ERK1/2 axis.

Lin et al. [32] observed that miR-26b-3p upregulation inhibited osteoblast differentiation via direct targeting ER-α, which was determined by dual luciferase assay. However, we found the relative mRNA and protein expression levels of ER-α did not show statistical difference between the group of miR-26b-3p overexpression and control group. This indicates that unlike pre-osteoblast cell, miR-26b-3p cannot directly regulate the ER-α in human chondrocytes. It is an interesting finding that though the significant difference on the relative mRNA and protein expression levels of ER-α was not observed between the group of miR-26b-3p overexpression and control group, we found the expression of p-ER-α was obviously downregulated in the group of miR-26b-3p overexpression compared with control group. Previous study [34] have confirmed that decreasing p-ERK1/2 activity can inhibit the levels of p-ER-α. Unlike Lin et al. finding [32], our study indicates that miR-26b-3p suppresses the function of ER-α via inhibiting the ER-α phosphorylation, not the relative mRNA and protein expression of ER-α in human chondrocytes. However, Börjesson et al. [45] found that cartilage-specific Era^−/−^ mice did not show significant differences on the body weight, femur length and Crown-rump compared with control mice. The present study indicates that miR-26b-3p upregulation of circulating plasma inhibit the chondrocyte proliferation and endochondral ossification dominantly via suppressing AKAP2/ ERK1/2 axis.

To further clarify the effect of miR-26b-3p overexpression in exosomes on cartilage growth and development, we overexpressed miR-26b-3p in vivo using exosomes or adenovirus. In vivo imaging revealed that exosomes highly aggregated on the lower limb growth plates after 48 h of the tail vein injection. In situ hybridization of rat femoral tissue sections further found that exosomes and miR-26b-3p were colonized in growth plate chondrocytes. Subsequent experiments further confirmed disorders of proliferation and endochondral ossification on growth plate cartilage of rats, just like in vitro experiment. After a month of miR-26b-3p overexpression in exosomes or adenovirus, the rats exhibited significantly lower femur, tibial, and overall height compared with the controls, indicating a short state phenotype. This study is the first to confirm that miR-26b-3p in plasma exosomes leads to disorders in proliferation and endochondral ossification of growth plate cartilage via inhibition of AKAP2/ERK1/2 axis, thereby inducing ISS.

## Conclusions

ISS is not a fatal condition. According to the requirements of medical ethics, growth plate chondrocyte samples during growth and development cannot be obtained. Hence, we cannot use growth plate chondrocytes of patients with ISS to verify our experimental results. We reported for the first time that plasma exosomes could inhibit the proliferation and endochondral ossification of human chondrocytes in ISS. Our in vivo findings revealed that exosome miR-26b-3p in circulating blood could be colonized in growth plates and could smoothly enter chondrocytes to regulate the AKAP2 axis, inhibiting proliferation and the endochondral ossification of growth plate chondrocytes and leading to short state phenotype. This study provides a new research direction for the etiology and pathology of ISS and a new idea for the biological treatment of ISS.

## Supplementary Information

**Additional file 1: Table S1.** Primers used for qRT-PCR analysis of mRNA levels.

**Additional file 2: Figure S1.** The flow chart demonstrates the detailed grouping information in rats.

**Additional file 3: Figure S2.** The miR-26b-3p plasmid (GV369) was successfully constructed. The sequence of miR-26b-3p plasmid or its mutated fragment.

**Additional file 4: Figure S3.** Western blot demonstrated that P62, ATG5, MTOR, NIN, MIBI and SERPINA1 expression was not downregulated upon miR-26b-3p overexpression.

**Additional file 5: Figure S4.** Overexpression of miR-26b-3p result in a short stature phenotype in rats. **A-D** Two months after miR-26b-3p overexpression, the body height and lengths of femur and tibia were significantly lowered compared to the blank control group. The data are presented as the mean ± SD. n = 3. Two groups were compared using *T*-test. *P < 0.05 vs. control.

## Data Availability

All data generated or analyzed during this study have been included in the article and the Additional information. The results of microarray analysis have been deposited to GEO (GEO Accession number: GSE193994).

## Abbreviations

ISS: Idiopathic short stature
OPN: Osteopontin
OCN: Osteocalcin
RUNX2: Runt-related transcription factor 2
CCK-8: Cell Counting Kit-8
ALP: Alkaline phosphatase
KEGG: Kyoto Encyclopedia of Genes and Genomes
GO: Gene ontology
ROC: Receiver operating characteristic
AUC: Area under the ROC curve
AKAP2: A-Kinase Anchoring Protein 2
ERK: Extracellular regulated protein kinases
ER-α: Estrogen Receptor alpha
