# Supplementary material for: Plasma exosome miRNA-26b-3p derived from idiopathic short stature impairs longitudinal bone growth via the AKAP2/ERK1/2 axis
Source: J Nanobiotechnology. 2023 Mar 16;21:94. doi: 10.1186/s12951-023-01849-8 (PMC10022307; doi:10.1186/s12951-023-01849-8)
Supplement: Supplementary file 1 — Additional file 1: Table S1. Primers used for qRT-PCR analysis of mRNA levels. [file 12951_2023_1849_MOESM1_ESM.docx]

Table S1

Primers used for qRT-PCR analysis of mRNA levels.

Target ID Primer sequence 5’-3’

AKAP2 F: ACTGACTAATCCGAGACCACC

R: TCAAAGGTGGAGGAGTGGGT

ERK1/2 F: CAACACCACCTGCGACCTT

R: CGTAGCCACATACTCCGTCA

ER-a F: CCATTGATAAAAACAGGAGGAA

R: TCACTGAAGGGTCTGGTAGGA

RUNX2 F: ACTTCCTGTGCTCCGTGCTG

R: TCGTTGAACCTGGCTACTTGG

COL10 F: GCAGCATTACGACCCAAGAT

R: CATGATTGAACTCCCTGAAG

OPN F: CCAGCCAAGGACCAACTACA

R: AGTGTTTGCTGTAATGCGCC

OCN F: GCACCACCGTTTAGGGCAT

R: CGTTCCTCATCTGGACTTTATTTTGC

GAPDH F: GGAGCGAGATCCCTCCAAAAT

R: GGCTGTTGTCATACTTCTCATGG

U6 F：CGCTTCGGCAGCACATATAC

R：AAATATGGAACGCTTCACGA

mir-26b-3p F：TGCGCCCTGTTCTCCATTACT

loop primer：GTCGTATCCAGTGCAGGGTCCGAGGTATTCGCACTGGATACGACAGCCAAGT

U6 F: CGCTTCGGCAGCACATATAC

R: AAATATGGAACGCTTCACGA
