# Supplementary figures and images for: Plasma exosome miRNA-26b-3p derived from idiopathic short stature impairs longitudinal bone growth via the AKAP2/ERK1/2 axis
Source: J Nanobiotechnology. 2023 Mar 16;21:94. doi: 10.1186/s12951-023-01849-8 (PMC10022307; doi:10.1186/s12951-023-01849-8)

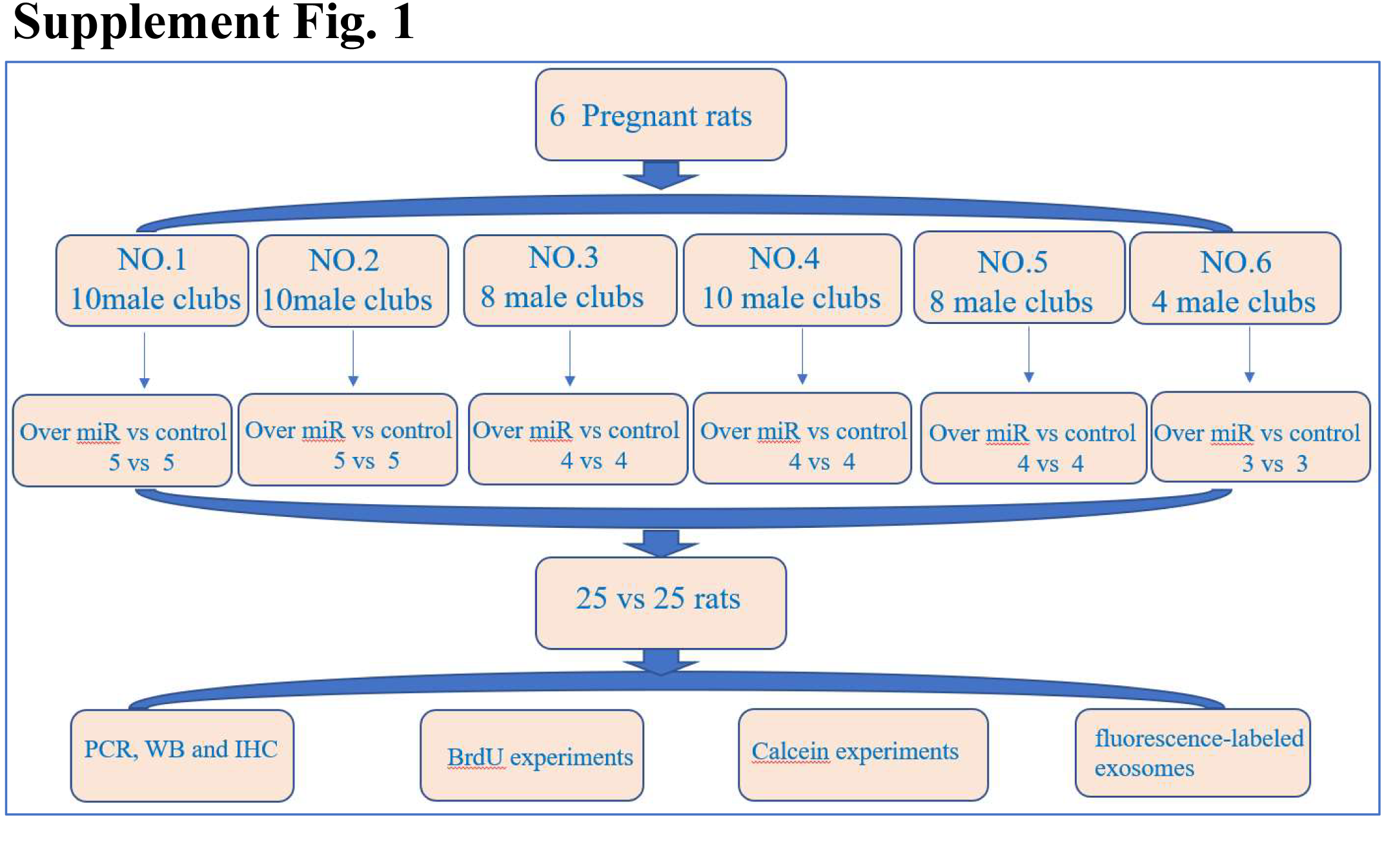

Supplement: Supplementary file 2 — Additional file 2: Figure S1. The flow chart demonstrates the detailed grouping information in rats. [file 12951_2023_1849_MOESM2_ESM.tif]

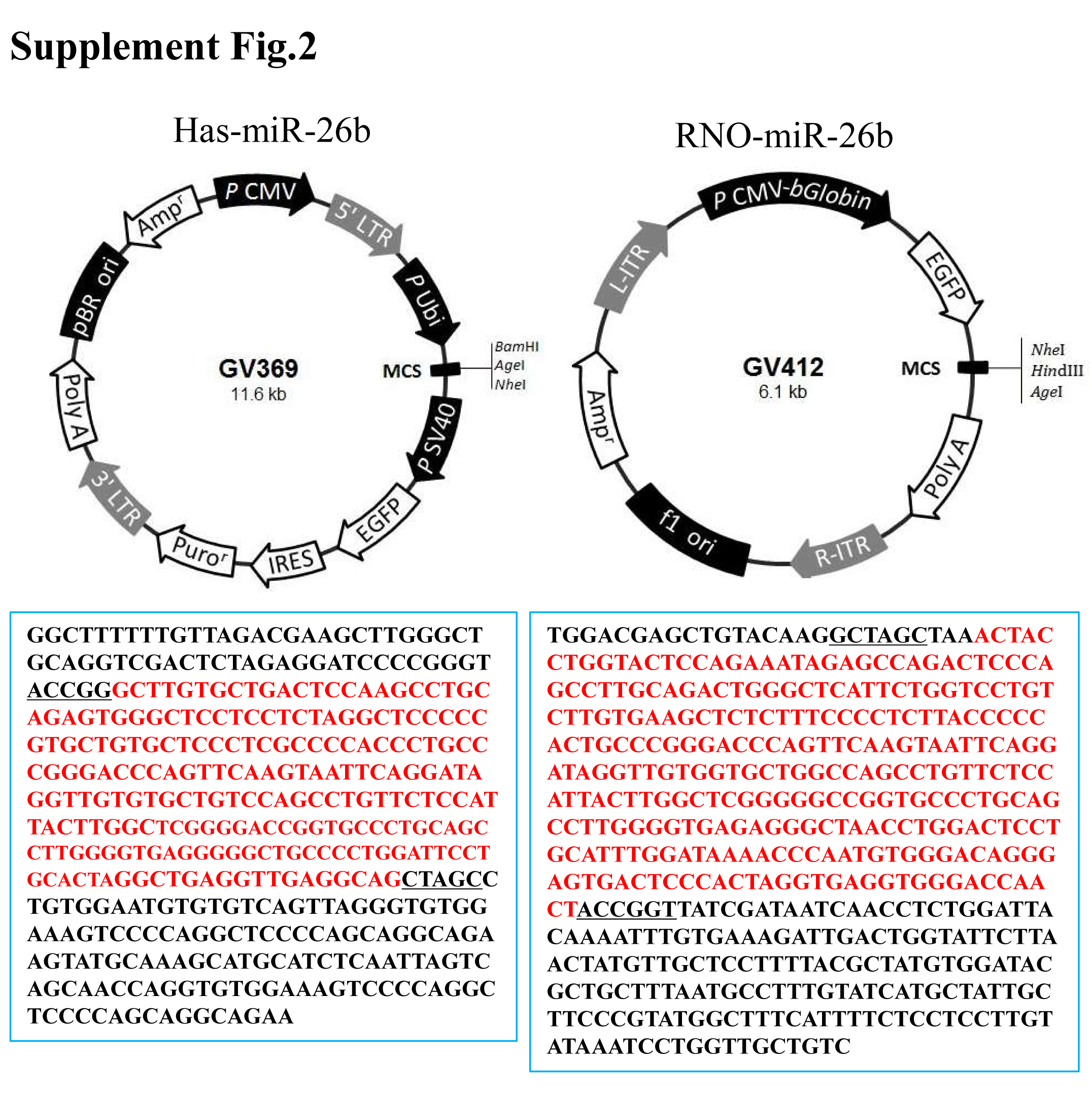

Supplement: Supplementary file 3 — Additional file 3: Figure S2. The miR-26b-3p plasmid (GV369) was successfully constructed. The sequence of miR-26b-3p plasmid or its mutated fragment. [file 12951_2023_1849_MOESM3_ESM.tif]

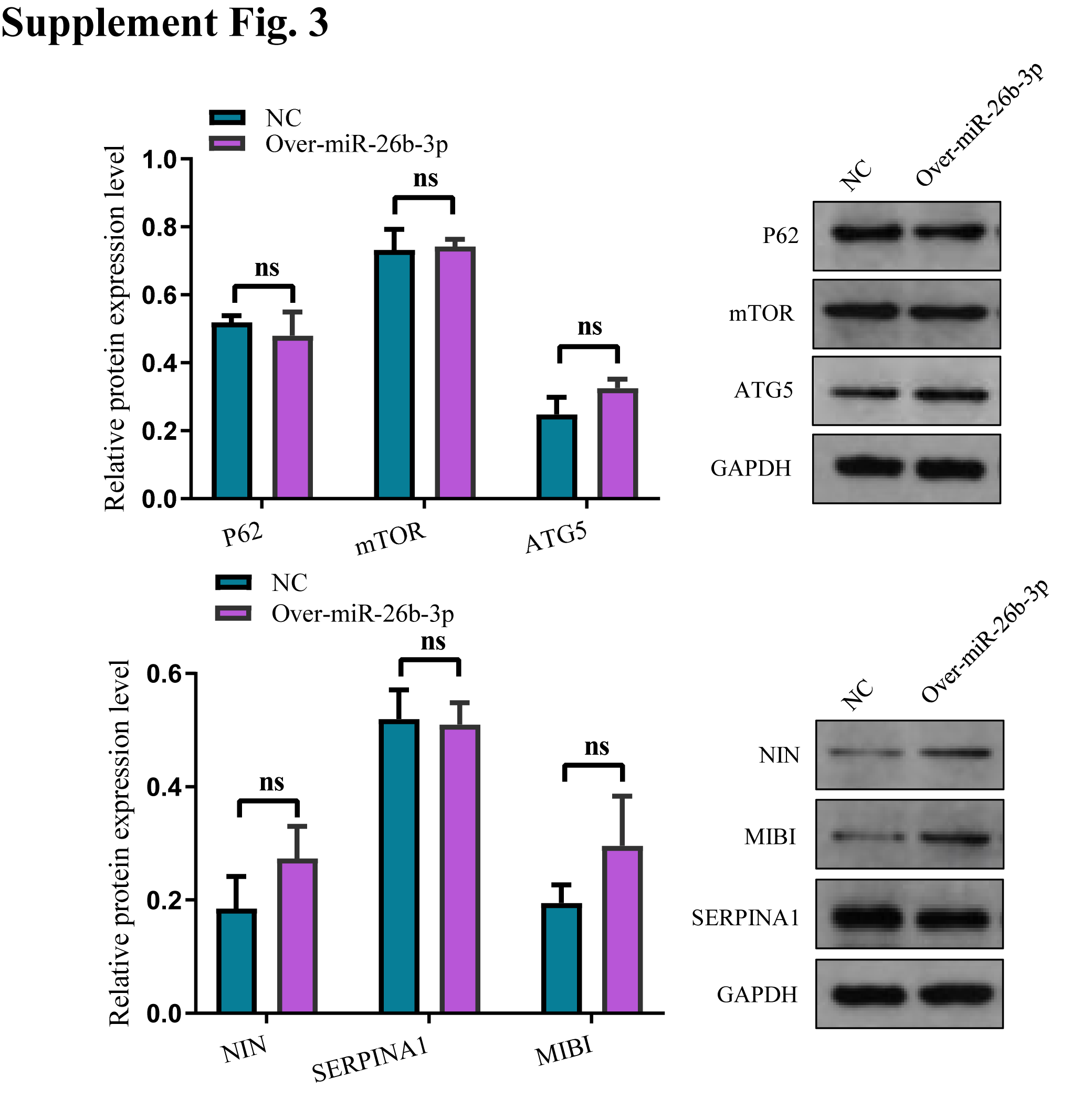

Supplement: Supplementary file 4 — Additional file 4: Figure S3. Western blot demonstrated that P62, ATG5, MTOR, NIN, MIBI and SERPINA1 expression was not downregulated upon miR-26b-3p overexpression. [file 12951_2023_1849_MOESM4_ESM.tif]

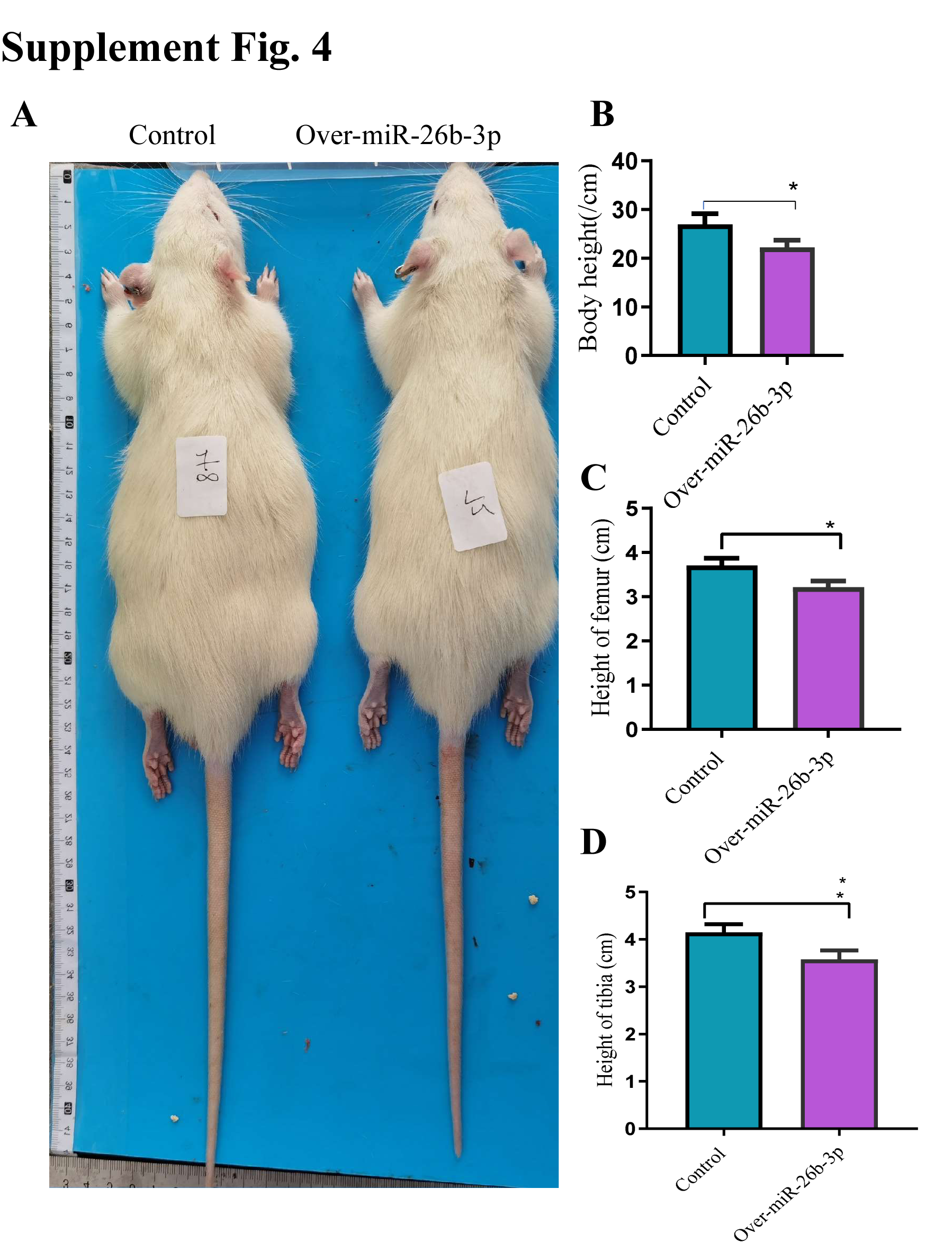

Supplement: Supplementary file 5 — Additional file 5: Figure S4. Overexpression of miR-26b-3p result in a short stature phenotype in rats. A-D Two months after miR-26b-3p overexpression, the body height and lengths of femur and tibia were significantly lowered compared to the blank control group. The data are presented as the mean ± SD. n = 3. Two groups were compared using T-test. *P < 0.05 vs. control. [file 12951_2023_1849_MOESM5_ESM.tif]
